# Programmed loading and rapid purification of engineered bacterial microcompartment shells

**DOI:** 10.1038/s41467-018-05162-z

**Published:** 2018-07-23

**Authors:** Andrew Hagen, Markus Sutter, Nancy Sloan, Cheryl A. Kerfeld

**Affiliations:** 1 Molecular Biophysics and Integrated Bioimaging Division, Lawrence Berkeley National Laboratory, 1 Cyclotron Road, Berkeley, CA 94720 USA; 2 MSU-DOE Plant Research Laboratory, Michigan State University, 612 Wilson Road, East Lansing, MI 48824 USA; 3 Department of Biochemistry and Molecular Biology, Michigan State University, 603 Wilson Road, East Lansing, MI 48824 USA

## Abstract

Bacterial microcompartments (BMCs) are selectively permeable proteinaceous organelles which encapsulate segments of metabolic pathways across bacterial phyla. They consist of an enzymatic core surrounded by a protein shell composed of multiple distinct proteins. Despite great potential in varied biotechnological applications, engineering efforts have been stymied by difficulties in their isolation and characterization and a dearth of robust methods for programming cores and shell permeability. We address these challenges by functionalizing shell proteins with affinity handles, enabling facile complementation-based affinity purification (CAP) and specific cargo docking sites for efficient encapsulation via covalent-linkage (EnCo). These shell functionalizations extend our knowledge of BMC architectural principles and enable the development of minimal shell systems of precisely defined structure and composition. The generalizability of CAP and EnCo will enable their application to functionally diverse microcompartment systems to facilitate both characterization of natural functions and the development of bespoke shells for selectively compartmentalizing proteins.

## Abstract (web summary)

Bacterial microcompartments are protein-bound organelles encapsulating segments of metabolic pathways. Here the authors functionalise shell proteins to facilitate facile purification and enable cargo encapsulation via covalent linkage.

## Introduction

Bacterial microcompartments (BMCs) are ensembles of enzymes enclosed in a semi-permeable proteinaceous shell, compositionally distinguishing them from the more familiar lipid-membrane bound organelles of eukaryotes. These compartments have been bioinformatically identified in the majority of bacterial phyla^1^ where they perform distinct, spatially separate metabolic functions allowing their hosts to fix carbon, as in the anabolic carboxysomes, or metabolize substrates like ethanolamine that generate volatile or toxic intermediates, as in the catabolic metabolosomes.

The shells of all characterized BMCs include a suite of cyclically symmetric protein building blocks which are hexagonal or pentagonal in shape and which together assemble into icosahedral bodies^2,3^. The hexagons are hexa- or trimeric oligomers of a conserved, single or tandem-duplicated protein domain (pfam00936) that constitute the facets of the shell. Pentamers formed by the pfam03319 domain occupy the vertices. Pores in the hexamer and trimer (BMC-H and BMC-T) subunits mediate passage of small molecules through the shells^4,5^. In all characterized BMCs, the presence of multiple BMC-H and/or BMC-T homologs, each with distinct pore-lining residues, indicate differences in selectivity of transport among these subunits. In contrast to their roles in molecular selectivity, the structural necessity of multiple homologs remains underexplored. Pentamers are a minor component of shells as they occupy only the 12 vertex positions and are not thought to play a role in transport, yet are critical to the shell’s function as a diffusive barrier^6^. The enzyme core of BMCs is assembled into the interior of shells through non-covalent interactions between short encapsulation peptides (EPs) on the cargo and the lumenal side of shell proteins^7–9^. A second mode of encapsulation involves piggybacking of EP-less enzymes via interactions with EP-harboring proteins^10^.

The genetic organization of BMC core and shell proteins into superloci^1^ and evidence of horizontal transfer among bacterial species^11,12^ denote BMCs as genetically encoded metabolic modules. Genetic transplantation of such loci has been shown to confer novel metabolic functions to the recipient allowing, for example, *Escherichia coli* to grow on 1,2-propanediol after transfer of the *pdu* operon from *Citrobacter freundii*^13^. Indeed, repurposing BMCs to encapsulate non-native pathways could allow metabolic engineers to improve titers by increasing enzyme stability, improving flux by concentrating intermediates and mitigating diversion of substrates to competing pathways, and sequestering potentially toxic or inhibitory intermediates^14–16^. Moreover, the nanoscale dimensions and greatly improved understanding of structure and assembly principles described recently^2^ suggest BMC shells are scaffolds fit for refactoring towards biomedical applications like polyvalent antigen display, drug delivery, and imaging—applications that have traditionally been the purview of other nanocompartments such as virus-like particles and ferritins^17,18^.

Hampering these ambitions are several technical challenges. Preparations of BMCs or BMC shells are laborious and typically result in low yields of heterogeneous particles (with some notable exceptions^19,20^). Targeting heterologous cargo to the lumen of shells using EPs that are derived from naturally occurring BMCs is inefficient, with western blotting often required to identify cargo in shell preparations^19–22^. EPs may also lead to aggregation, potentially compromising enzyme activity^23^, and the binding site of EPs is unknown so cargo loading in shells is not readily programmable. Furthermore, there is no disassembly/reassembly methods developed which would allow in vitro encapsulation of cargo.

Here, through rational modifications to shell proteins in our model BMC system HO (from *Haliangium ochraceum*), we report the development of the CAP and EnCo methods. Complementation-based Affinity Purification (CAP) enables facile screening of assembly and rapid purification of shells. We apply this method to demonstrate a remarkable plasticity of subunit composition in HO shells. Encapsulation via Covalent-linkage (EnCo) is used to robustly program multiple cargo species into shells at predictable ratios both in vivo and ex vivo, enabling the encapsulation of heterologously produced cargo and abiotic materials. Finally, we apply a fluorescence-based probe to assess the permeability of our engineered shells and find they maintain their barrier functionality against macromolecular species while permitting ingress of a small molecule—likely through the pores of one or more shell proteins. These methodological advances should be applicable to other BMC systems, simplifying and accelerating assembly and permeability studies as well as elevate BMC shells to the pantheon of well-characterized and engineerable nanoparticles, enabling their application towards metabolic engineering and biomedical applications.

## Results

### Shells lacking pentamers can be capped and affinity purified

To obtain a crystal structure of synthetic BMC shells it was necessary to supplement shells with additional copies of the pentamer-forming proteins. This suggests substoichiometric production of pentamer during expression of the *Haliangium ochraceum* (HO) shell synthetic operon^2^. Previously, we had observed that facets assembled into three-dimensional particles independent of the presence of pentamers^20^; taken together, these findings suggested a strategy for producing shells lacking vertices that could be subsequently capped and purified. We hypothesized that appending a high affinity, specific tag such as the Strep-II sequence^24^ to the pentamer (P_SII_) would enable pull-downs of shells after capping. Additionally, affixing the ten residues, 1.2 kDa Strep-II tag to the pentamer would result in a size shift allowing it to be resolved from the 10.1 kDa hexamer protein via SDS-PAGE analysis. These combined properties would enable rapid purification and analysis of shells by obviating the need for laborious and technically demanding density gradient ultracentrifugation and ion-exchange chromatography steps.

**Fig. 1:**
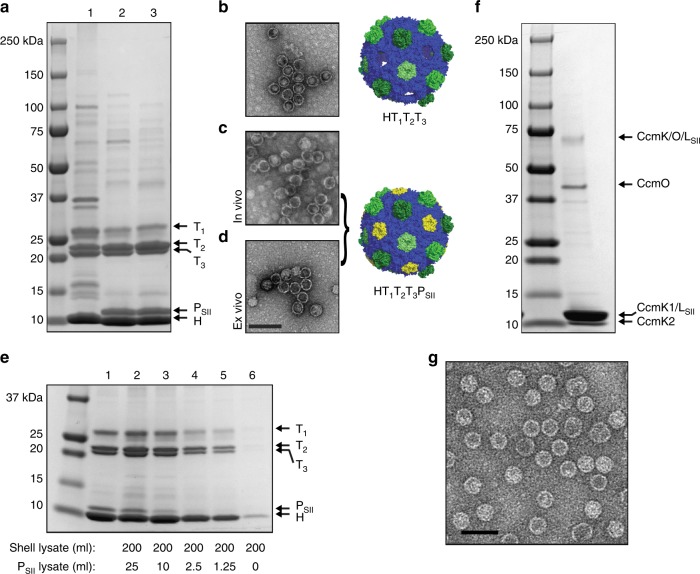
Comparison of different shell preparation methods. **a** SDS-PAGE analysis of HO shell preparations. Lane 1: Classic, Lane 2: in vivo capped, Lane 3: ex vivo capped. Shell protein identities indicated by arrows; loading normalized to A280 readings. **b**–**d** Negative stain TEM micrographs of three different shell preparations and corresponding structural models. Scale bar = 100 nm. **e** SDS-PAGE analysis of eluates from pentamer titration experiment. Lane 1: Ex vivo capped shells from Fig. 1a, Lanes 2–6: Titration of pentamers with equivalent volumes of each culture used in mixing experiments expressed in milliliters. **f** SDS-PAGE analysis of *Halo* carboxysome shell preparation using affinity purification. **g** Negative stain TEM micrographs of *Halo* carboxysome shell preparation. Scale bar = 50 nm. Results presented are representative of two independent biological replicates for **a**–**d**, **f**, and **g**. Findings in figure (**e**) recapitulate pilot experiments in which a subset of specified lysate ratios were used

We validated this hypothesis by comparing classically^2,20^ prepared HO shells comprised of a single hexamer and three trimer homologs (HT_1_T_2_T_3_ shells; uncapped shells were used because the co-migrating pentamer and hexamer bands preclude fair comparison between samples) to capped HT_1_T_2_T_3_P_SII_ shells prepared by two methods. In the first method, which recapitulates normal in vivo assembly of shells, P_SII_ proteins were co-expressed from a secondary plasmid in the same strain, and then purified via a StrepTrap column. In the second method, ex vivo capping, clarified lysates of the HT_1_T_2_T_3_ expressing strain and a separate strain expressing P_SII_ were mixed and after a brief incubation, purified via StrepTrap. Excess P_SII_ proteins and trace contaminants were subsequently removed via anion-exchange chromatography (see Methods for details and Supplementary Figs. 1–3 for complete purification schemes and tracking). Figure 1a shows that HT_1_T_2_T_3_P_SII_ shells are obtained when either the in vivo (lane 2) or ex vivo (lane 3) capping/pull-down methods are employed according to SDS-PAGE analysis. We observe no morphological changes to our affinity-purified shells (Fig. 1b–d), indicating that the Strep-II-tag is well-tolerated. It is unclear why there are subtle differences in the abundance of contaminants between lanes 2 and 3 (e.g., the band migrating between 50 and 75 kDa), however these observations are reproducible and may arise from the different plasmid content of the strains used (a double-transformed strain vs. a mixture of two singly-transformed strains). Nevertheless, the classically prepared shells are ~70% pure whereas both CAP-purified shell preparations are >95% pure and the entire protocol from cell pellets to shells can be performed in a matter of hours. In addition, the final yield of shells for the different methods is similar, ranging from 0.40 to 0.55 mg purified shell per gram cell pellet. Control reactions using a pentamer variant without the Strep-II tag fail to appreciably recover shells during the ex vivo CAP protocol, indicating that the method is dependent on specific interactions with the affinity resin (Supplementary Fig. 3c).

Analysis of the initial ex vivo capping experiment suggested that at the 1:2 (v/v) mixing ratio of pentamer lysate to shell lysate used, the pentamers are present in stoichiometric excess (i.e., greater than 12 pentamer subunits per shell particle). This is evidenced by a substantial peak comprised primarily of pentamer protein which elutes prior to the capped shells during the anion-exchange chromatography run (Supplementary Fig. 3b). While we cannot be certain at this stage that every vertex is occupied when pentamers are in such excess, we nonetheless performed a pentamer titration to determine whether putative substoichiometric amounts of pentamer provide sufficient avidity to pull-down the (presumably) partially capped shells. Equal volumes of shell lysates were mixed with a range of pentamer lysate amounts (as low as 1:160 pentamer to shell volumetric mixing) and CAP-purified. The eluates were subjected to SDS-PAGE analysis, this time without anion-exchange polishing steps in order to preserve the relative ratios of all eluted proteins. As shown in Fig. 1e, decreasing pentamer to hexamer band intensities in tandem with decreased pentamer loading indicate that sub stoichiometric amounts of pentamer are still able to recover shells and overall impact on shell yield is modest above a certain pentamer loading threshold. We attribute the trace amounts of shells recovered in the no-pentamer control lane to incomplete elution of pentamers and/or shells from previous StrepTrap chromatography runs. Together, these results indicate that partially capped shells maintain sufficient avidity to be CAP-purified—a property that may be applied toward rapid purification of shells that can still be loaded with cargo ex vivo through the remaining pentamer vacancies and then fully capped.

Finally, we show that other shell systems are amenable to affinity-based purification by appending a Strep-II tag to the pentameric CcmL protein in a synthetic operon used to produce beta-carboxysomal shells from *Halothece* sp. PCC 7418^19^ (*Halo* hereafter). As with the in vivo CAP described above, proteins were heterologously expressed in *E. coli* and purified by StrepTrap and anion-exchange. Figures 1f, g reveals that highly pure, morphologically homogeneous particles are obtained via affinity purification. In the native systems, the absence of CcmL typically results in elongated carboxysomes; the presence of pentamer seems to be required for the termination of facet growth^10,25^. Accordingly, uncapped synthetic carboxysome shells cannot be complemented *in trans* as in the ex vivo CAP method used for HO shells. Nevertheless, the rapid isolation of BMCs of a completely different type suggests the method may be broadly applicable to other shells systems.

### CAP facilitates screening of shell formation to define a minimal shell

Recent work in our lab^2^ revealed HO shells to be T = 9 icosahedra with one of the three possible BMC-T (T_1–3_) proteins occupying the central position of each facet. Due to high shape complementarity between the trimers despite significant primary sequence divergence, we predicted that the trimers would be interchangeable. We therefore employed CAP to screen for the potential formation of shells comprised of the hexamer, one of the three trimers, and the pentamer protein. Shells were produced by expression from three plasmids each bicistronically expressing the hexamer and a single trimer. Because we were employing this method as a facile screen for shell assembly, we omitted subsequent anion-exchange polishing steps. Consequently, by ensuring P_SII_ protein is in stoichiometric excess and equally applied to each HT_n_ lysate, densitometric analysis of the P_SII_ to hexamer band-intensity ratios gives a crude measure of relative shell yields (SDS-PAGE samples are loaded based on normalized A280 values).

**Fig. 2:**
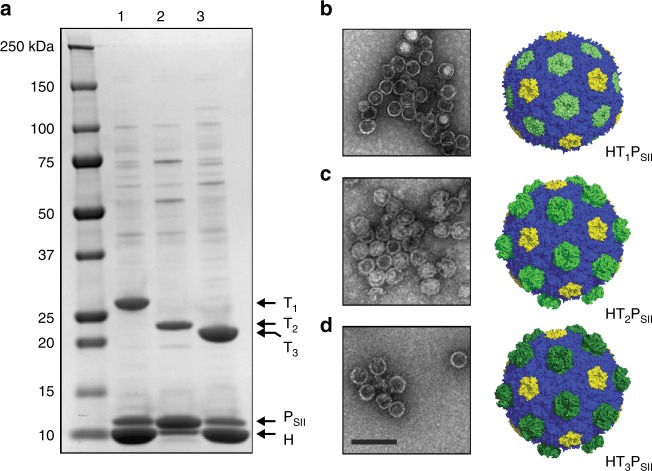
Comparison of different minimal shells. **a** SDS-PAGE analysis of crude shell preparations. Lane 1: HT_1_P_SII_ Lane 2: HT_2_P_SII_ Lane 3: HT_3_P_SII_. Shell protein identities indicated by arrows. **b**–**d** Negative stain TEM micrographs of three minimal shell preparations and corresponding structural models. Scale bar = 100 nm

As shown in Fig. 2a, expression of any single BMC-T domain with BMC-H is sufficient to form shells as assayed by CAP. To our knowledge, shell formation from just a single species each of hexamer, trimer and pentamer proteins has not previously been observed. Because identical trimers occupy each facet in these minimal shells, the method provides a means of isolation of BMC shells that are molecularly defined icosahedra. Interestingly, the P_SII_ to BMC-H ratios reveal notable differences between the band intensities of the three shell preparations. Whereas shells comprised of just T_1_ and T_3_ trimers (HT_1_P_SII_ and HT_3_P_SII_, respectively) show similar pentamer:hexamer ratios (lanes 1 and 3), the yield of HT_2_P_SII_ shells is markedly reduced as indicated by a lower hexamer:pentamer band-intensity ratio. Whether this is attributable to the impact a particular BMC-T has on shell assembly efficiency or is merely an artifact of differential expression/stability between the trimers remains undetermined. We note no major morphological differences in these minimal shell preparations, further reinforcing the notion that each individual trimer serves identical structural roles within the context of the shell architecture, despite their presumed differences in permeability (Fig. 2b, d).

### EnCO enables robust encapsulation of cargo

To date, published encapsulation methods for heterologous cargo have been inefficient^19–22^. We sought a synthetic route to encapsulation that circumvents the shortcomings of EPs by using the SpyTag/SpyCatcher split bacterial adhesin system^26^. This technology relies on genetically fusing a 9 kDa SpyCatcher domain to one protein partner and a 1.4 kDa (13 residue) SpyTag to another protein partner. When the SpyCatcher and SpyTag polypeptides encounter one another, an isopeptide bond autocatalytically forms between them, covalently linking the two partner proteins. We reasoned that a SpyCatcher or SpyTag localized to the lumen of a BMC shell would serve as a defined docking site for cargo protein tagged with SpyTag or SpyCatcher (respectively).

**Fig. 3:**
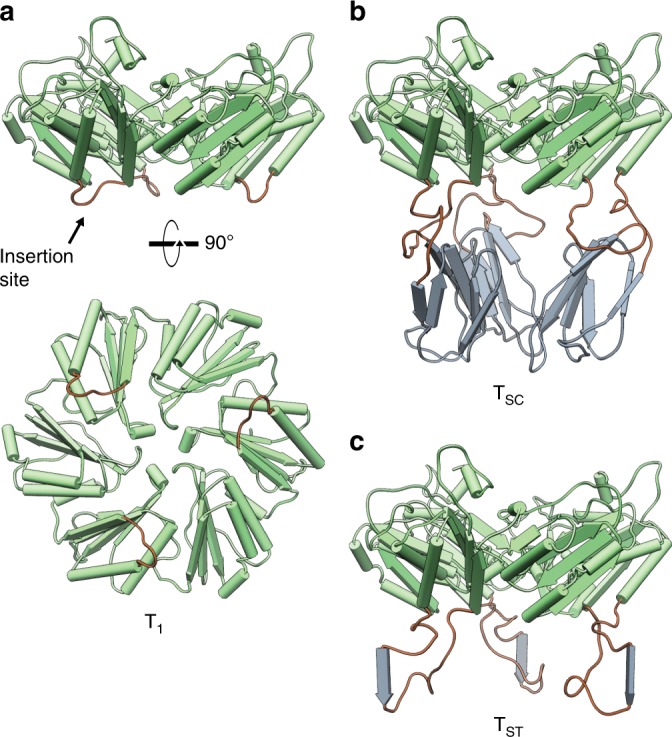
Molecular models of T_1_, T_SC_ and T_ST_ subunits. **a** Model of T_1_ (wt) (PDB: 5DIH) as viewed from side of shell (top) and lumen (bottom). **b** and **c** Models of T_SC_ and T_ST_ (respectively) viewed from the side. Flexible coil regions colored in brown; SpyCatcher and SpyTag regions colored in gray

T_1_ was chosen as a target for insertional mutagenesis of the SpyTag and SpyCatcher sequences. As shown in Fig. 3a, a region was identified on the lumenal side between alpha-helix two and beta-strand four, which is not predicted to make any contacts with neighboring shell proteins and which lies ~24 Å from the subunit’s central pore (the entire subunit is ~70 Å edge-to-edge). This spacing from the threefold symmetry axis of the subunit mitigates the possibility of steric clashes from inserted/conjugated domains which could prevent the assembly of individual protomers into the trimeric subunit and/or result in incomplete cargo conjugation. SpyCatcher and SpyTag, flanked by six residue glycine-serine linkers, were inserted into T_1_ to create T_SC_ and T_ST_ constructs, respectively (Fig. 3b, c).

The wild-type T_1_ was replaced with the T_SC_ and T_ST_ mutants in plasmids that express the full complement of trimers (pHT_1_T_2_T_3_) as well as the minimal shell producing pHT_1_ plasmid to create, respectively, pHT_SC_T_2_T_3_/pHT_SC_ and pHT_ST_T_2_T_3_/pHT_ST_. SpyTag and SpyCatcher was appended to the N-terminus of the cyan fluorescent protein mTurquoise2^27^ and cloned into a compatible and orthogonally inducible plasmid with a C-terminal hexahistidine tag (_ST_cfp).

**Fig. 4:**
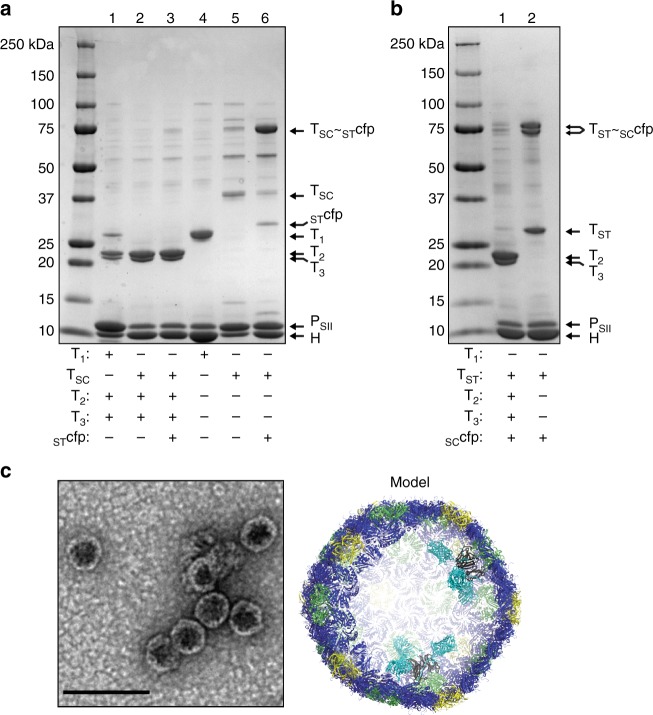
SDS-PAGE and electron micrographs of various shell preparations. **a**, **b** SDS-PAGE of shell preparations. Composition of shell preparations given in tabular form below each lane. **c** Negative stain TEM of HT_SC_~_ST_cfpP_SII_ shell preparation (lane 6 in figure **a**) and cutaway model of shells (cfp rendered in turquoise, not completely functionalized for clarity). Results presented are representative of at least two independent biological replicates of each sample preparation

Shell and cargo plasmids were co-transformed and shell expression was induced either with or without simultaneous induction of _ST_cfp cargo. CAP was then used to isolate the resultant shells. SDS-PAGE analysis of unpolished eluates is shown in Fig. 4a. In contrast to the wild-type shells in lane 1 in which the wild-type T_1_ protein is detected at its normal relative abundance, the T_SC_ is undetectable in the putative HT_SC_T_2_T_3_P_SII_ shells. However in lane 3, the fused T_SC_~_ST_cfp trimer, which migrates at ~75 kDa, is observed—likely due to the increase in the protein mass upon conjugation to cargo allowing the fusion to exceed the stain’s limit of detection. This would suggest that the T_SC_ protein is at a modest competitive disadvantage for integration into shells compared to wild-type T_1_ and T_2_/T_3_ and/or could result from reduced solubility of T_SC_ relative to T_1_. Nevertheless, these HT_SC_~_ST_cfpT_2_T_3_P_SII_ shells exhibit normal morphology by microscopic analysis and have a fluorescence emission spectrum consistent with mTurquoise2 (Supplementary Figs. 4a, b), thus proving that cargo-fused T_SC_ can integrate into shells without any gross morphological disruption. Furthermore, whereas EP-based encapsulation in the HO shell system (among others) requires western blotting to detect cargo, the detection of loaded cargo by coomassie stain alone represents a significant improvement over existing methods.

Minimal shells harboring only the T_SC_ subunit have more cargo binding capacity due to the absence of competition for the trimer subunit position with T_2_ and T_3_ proteins—a theoretical 60 copies of cargo could be recruited by the twenty trimeric subunits. This was confirmed by characterizing cargo loading using the pHT_SC_ plasmid and results are shown in Fig. 4a lanes 4–6. Lane 5, in which expression of cargo is not induced, now reveals the presence of T_SC_ as expected (lest no shells be formed) and lane 6 shows the T_SC_ band is nearly completely converted to the cargo-fused T_SC_~_ST_cfp form in these shell preparations. Interestingly, a small amount of free _ST_cfp co-purifies with the shells (lane 6) which could be a result of stochastic encapsulation or proteolytic deconjugation from T_SC_. Western blotting against the hexahistidine tag present in _ST_cfp confirms the identity of these proteins and reveals that the faint band at ~75 kDa in lane 5 is a contaminant and not T_SC_~_ST_cfp which could result from leaky expression of the _ST_cfp in the absence of inducing agent (Supplementary Fig. 4c).

When SpyTag is inserted into T_1_ and SpyCatcher-fused to cargo (i.e. the inverse arrangement), similar behavior is observed in the context of full shells: the T_ST_~_SC_cfp fusion is detected but not at the relative abundance expected in wild-type HT_1_T_2_T_3_P_SII_ shells (Fig. 4b lane 1). However, in comparison to the minimal HT_SC_~_ST_cfpP_SII_ shells in Fig. 4a lane 6, a significant fraction of the T_ST_ subunit remains unconjugated to the _SC_cfp cargo (Fig. 4b lane 2). This could indicate that the T_ST_ is less efficient at recruiting cargo compared to T_SC_ and/or that SpyCatcher-fused cargo is sterically frustrated from productive binding to the T_ST_ subunit. A final, non-mutually exclusive scenario is that shells form prior to cargo recruitment and the larger _SC_cfp (42.7 kDa) is less able to transit the pentamer vacancies compared to _ST_cfp (29.9 kDa). HT_ST_~_SC_cfpP_SII_ shells are obtained at levels similar to HT_1_T_2_T_3_P_SII_ shells (0.40 mg per gram of cell pellet) while HT_SC_~_ST_cfpP_SII_ shells incur a significant reduction in yield (0.04 mg per gram cell pellet), though purity is comparable after anion-exchange chromatography is performed (Supplementary Fig. 5a). Finally, we calculated the number of cfp cargo proteins per shell using fluorescence intensity values and find that HT_SC_~_ST_cfpP_SII_ shells have ~73 copies per shell and HT_ST_~_SC_cfpP_SII_ shells have ~58 copies per shell (Supplementary Fig. 5b). These numbers are in reasonable agreement with theoretical estimates based on percent conversion of the trimers to their respective conjugates as observed in SDS-PAGE analysis.

Based on the assembly principles and structure of HO shells reported by Sutter et al.^2^, the minimal shells in lanes 4–6 harbor 20 trimer subunits, nearly all of which are conjugated to cargo in the HT_SC_~_ST_cfpP_SII_ shell preparations according to the aforementioned SDS-PAGE and fluorescence analysis. This should increase the apparent shell thickness and indeed, examination of transmission electron micrographs of the minimal shell compositions depicted in Fig. 4a lane 6 reveal notable differences in shell appearance: cargo-loaded shells appear thicker at ~6.2 nm and more stippled compared to HT_1_P_SII_ shells (Fig. 2b, ~4.7 nm; measurements are mean of *n* = 3 discrete shells) as well as empty HT_SC_P_SII_ shells (Supplementary Fig. 6; see Supplementary Fig. 7 for shell measurements). Together, these findings validate EnCo as a robust method for encapsulation of cargo into morphologically undisrupted shells using trimer subunits functionalized with molecular traps.

### Multiple cargo proteins can be loaded ex vivo at defined ratios

The in vivo encapsulation results do not allow us to distinguish between two non-mutually exclusive scenarios: T_SC/ST_~cargo conjugation occurs prior to integration into proto-shells, or empty shells form first and cargo then conjugates to the trimer subunits after entry through the pentamer holes. The pentamer vacancies are ~47 Å diameter and can accommodate mTurquoise2 (Supplementary Fig. 8a). We tested whether cargo could be loaded into pre-formed shells ex vivo in biological duplicates by mixing clarified lysates of strains expressing HT_SC_ shells with clarified lysates containing the previously described _ST_cfp as well as SpyTagged yellow fluorescent protein, SYFP2^28^ (_ST_yfp) at varying volumetric ratios. These two fluorophores are spectrally well-matched as a Förster resonance energy transfer (FRET) pair^27^ and therefore their co-localization is detectable spectrophotometrically. Expression under identical conditions in separate strains results in the same abundance of the _ST_cfp and _ST_yfp proteins in whole cell lysates (Supplementary Fig. 8b).

**Fig. 5:**
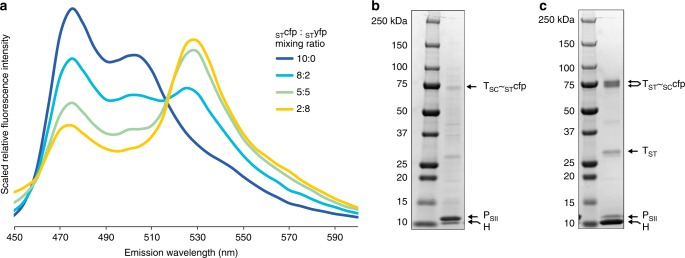
Fluorescence spectra and SDS-PAGE analysis of shells containing ex vivo programmed cargo. **a** Scaled emission spectra (excitation: 405 nm) of programmed cargo. _ST_yfp-only trace (0:10) was not plotted—in the absence of a FRET donor, the fluorescence signal is negligible. **b** SDS-PAGE analysis of _ST_cfp (10:0) programmed shells. **c** SDS-PAGE analysis of _SC_cfp programmed shells. Results are representative of two independent biological replicates

After cargo loading, the mixtures were subjected to CAP and analyzed spectrophotometrically; flow-through fractions which contain unencapsulated _ST_cfp/_ST_yfp were also collected for comparison. Shells were excited at 405 nm (a wavelength capable of _ST_cfp excitation with negligible excitation of _ST_yfp) and emission spectra were collected from 450 to 600 nm. As shown in Fig. 5a, increasing the proportion of _ST_yfp in the mixture attenuates the emission of _ST_cfp (emission maximum 474 nm) with a concomitant increase in emission from _ST_yfp (emission maximum 527 nm) indicating FRET and co-localization of the two fluorophores. Comparison of the fluorescence intensities of the two fluorophores in both the shell preparations and flow-through fractions corroborate these emission scan data (Supplementary Fig. 8d). These data demonstrate that _ST_cfp and _ST_yfp co-localization is mediated by co-encapsulation within HT_SC_ shells and that multi-cargo relative abundances can be easily programmed via ex vivo encapsulation—an important consideration for optimizing flux through multi-enzyme cascades and which is not readily achievable in vivo without laborious optimization of cargo expression levels. Finally, the encapsulation efficiency of ex vivo cargo loading for these HT_SC_~_ST_cfpP_SII_ shells was assessed by SDS-PAGE analysis along with the inverted architecture (HT_ST_~_SC_cfpP_SII_ shells). As shown in Fig. 5b, c, the amount of unconjugated T_SC_ and T_ST_ is similar, if not lower compared to the in vivo encapsulation experiments (Fig. 4a lane 6 and 4b lane 2). Because these ex vivo-loaded shells are formed prior to the introduction of cargo, this indicates that the observed relative encapsulation efficiency of the two EnCo architectures is not due to the different sizes of cargo transiting through the pentamer gaps. Control experiments in which incompatibly-tagged cargo was tested for encapsulation (e.g., using _SC_cfp with HT_SC_ shells) reveals non-specific stochastic encapsulation is minor (Supplementary Fig. 8e).

### Interrogation of HO shell permeability using a FRET-based sensor

**Fig. 6:**
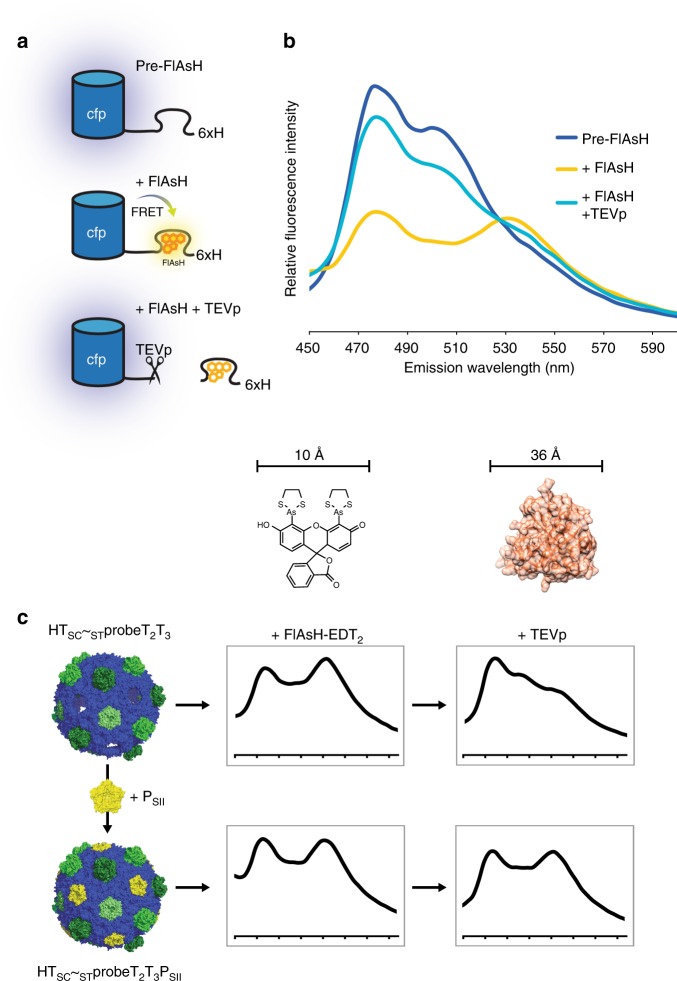
Probe properties and permeability assay of uncapped and capped shells. **a** Schematic of probe behavior in presence of FlAsH and TEV protease. **b** Fluorescence emission spectra (excitation: 405 nm) of unencapsulated probe. **c** Emission spectra (450–600 nm) of encapsulated probe in uncapped and capped shells, in the presence of FlAsH and TEV protease. Axis numbers omitted for clarity; tick marks correspond to numbers in **b**. Results are representative of two independent technical replicates. Bacterial microcompartments are protein-bound organelles encapsulating segments of metabolic pathways. Here the authors functionalize shell proteins to facilitate facile purification and enable cargo encapsulation via covalent linkage

In comparison to storage-based nanocompartments like viruses and ferritins^29^, BMC function depends on selective permeability to an array of molecules—the identities of which depend on the particular native physiological function of a given BMC (recently reviewed in ref.^30^). The uncapped HO shells admit macromolecular species yet should prevent their entry/egress when fully capped. To test this behavior and confirm that CAP results in fully complemented shells, we developed a FRET-based permeability probe and apply it towards elucidating the permeability of HO shells to protein and small molecule size scales. The tobacco etch virus protease (TEVp) cleavage site^31^ followed by a six-residue tetracysteine motif was inserted between the C-terminus of mTurquoise2 and the hexahistidine tag of the _ST_cfp construct to create the SpyTagged permeability probe, _ST_probe. Tetracysteine motifs tightly and specifically bind conditionally fluorescent biarsenical dyes such as the fluorescein-derived FlAsH reagent^32^. At 664.5 Da and ~10 Å at its narrowest point, the FlAsH reagent is in a similar size regime as cofactors like NADPH (744.4 Da) and therefore may be admitted through some subunit pores—likely one or more trimers, as the hexamer’s ~6.8 Å pore is too small to accommodate the FlAsH reagent^5,33,34^. The spectral properties of FlAsH are similar to SYFP2; binding to _ST_probe positions the fluorophore within the Förster distance of mTurquoise2 and therefore this event can be detected spectrophotometrically. The spectral shift can subsequently be reversed by proteolytic removal of the labeled tetracysteine motif using TEVp. _ST_probe’s design and spectrophotometric behavior in response to incubation with FlAsH and TEVp when unencapsulated are depicted in Fig. 6a, b. Because TEVp also removes the C-terminal hexahistidine tag from the probe, cleavage can also be detected using western blotting against this epitope. SDS-PAGE analysis of the final probe preparation and western blotting confirm that, as expected, the BMC-P protein itself has no inhibitory activity on TEVp (Supplementary Fig. 9a, b).

We performed a classical shell preparation of _ST_probe-loaded, uncapped shells (HT_SC_~_ST_probeT_2_T_3_). Addition of the FlAsH reagent to the shells results in a fluorescence emission shift which is reverted upon TEVp addition (Fig. 6c, top row), recapitulating the behavior of unencapsulated probe. This demonstrates, as expected, that FlAsH and TEVp (~36 Å in diameter) can enter the shells and proteolyze the encapsulated probe. We next show that shells pre-capped via incubation with purified pentamer (HT_SC_~_ST_probeT_2_T_3_P_SII_) still undergo a spectral shift when FlAsH is added, yet show no reversion in spectral emission upon addition of TEVp (Fig. 6c, bottom row). Exclusion of TEVp from these capped shells is confirmed by western blot analysis (Supplementary Fig. 9d). We conclude that addition of the pentamer to uncapped shells is necessary and sufficient to preclude the entry of TEVp, though interestingly, these capped shells remain permeable to the FlAsH reagent, indicating it may be entering through the pores of one or more subunits. By leveraging EnCo for encapsulation of a permeability probe, these data indicate that CAP yields fully complemented shells with no unoccupied pentamer positions.

## Discussion

In this study, we have elaborated the function of bacterial microcompartment shell proteins through the introduction of affinity handles and molecular traps. Complementation-based affinity purification (CAP) enables rapid purification of shells and by extension, screening for the formation of particles comprising varying subunits. Encapsulation via covalent-linkage (EnCo) allows facile programming of cargo composition expressed in cis and in trans. Using a permeability probe, we unambiguously demonstrate that shells capped *in trans* are fully complemented barriers against the transit of a macromolecular species (TEV protease) and yet admit the small molecule, FlAsH—likely through one or more of the distinct types of pores found in the shell proteins.

Previous work has shown that other BMCs such as the carboxysome can form without pentamers, though aberrant structures were observed microscopically in cells^6,10,25^. In contrast, we observe no structural polymorphisms in HO shell preparations when pentamers are absent. Moreover, the isolation of morphologically undisrupted minimal shells, each harboring a single trimer species, implies redundancy at the structural level while differing pore and stacking configurations implicate non-redundant functions in selective molecular transport across the shell. The ability to form uncapped shells enables the encapsulation of cargo ex vivo. As demonstrated, this allows multiple cargo species (which need not be expressed in the shell host) to be introduced into the shells at programmed ratios. Trapping SpyTag/SpyCatcher-fused cargo in shells via EnCo is simple and efficient; however in principle any species able to transit the 47 Å diameter pentamer gap could be captured via co-incubation and capping. Such stochastic encapsulation would be of particular use in encapsulating molecules which cannot be tagged (e.g., acyl-CoA substrates), or even wholly abiotic materials.

Although there is a report of affinity enrichment of bacterial microcompartments^35^, the resultant particles were heterogeneous and morphologically aberrant. This may be attributable to appending hexahistidine tags to multiple shell proteins which may sterically frustrate proper assembly. In contrast, the lack of any pentamer-pentamer subunit interactions in assembled shells advantages the use of this building block for embelishments and we have demonstrated that very few Strep-II tags per particle provide sufficient avidity to pull-down highly pure shells. The isolation of beta-carboxysomal (*Halo*) shells demonstrates that this affinity pull-down strategy is applicable to other microcompartments that frequently exhibit morphological heterogeneity. Given the homology among the shell proteins of all BMCs, we expect that this method, after identifying specific combinations of subunits to form synthetic shells, will be generally applicable to other BMC systems.

The observations by us and others that encapsulation peptides are often ineffective at recruiting heterologous cargo to the lumen of BMC shells motivated us to pursue alternative means of encapsulation. Strategies for nanoparticle encapsulation such as engineering complementary electrostatics have proven useful for other nanocontainers such as lumazine synthase^36^. However, this approach is not targeted and may non-specifically package resident charged molecules (e.g., nucleic acids) when performed in vivo. The SpyTag/SpyCatcher system has been used for encapsulation in other nanocompartments such as MS2 viral capsids and excitingly, encapsulated enzymes were shown to be more robust^37^. However MS2 viral capsids are composed of a single protein type and therefore lack any potential for compositional plasticity which may decrease their utility. We believe that shared architectural features and structurally homologous insertion sites will allow translation of the SpyCatcher/SpyTag system to other BMC types. Moreover, despite the demonstrated superiority of EnCo to existing technology, improvements may be realized with additional protein engineering. For example alternatives to the currently employed poly-G/S linkers that flank SpyTag/SpyCatcher could improve solubility, cargo recruitment and integration into shells. Additionally, because expression and shell formation dynamics remain unexplored, in vivo metabolic engineering applications will require empirical testing of induction timing and strength to optimize loading efficiency.

While numerous studies have demonstrated that the permeability of BMCs can be altered via rational mutagenesis of pore-lining residues, or even whole-sale swapping of subunits from one BMC system to another^38–40^, assessing BMC permeability to a given small molecule is non-trivial, much less elucidating the permeability of specific subunits to said molecule. Our fluorescence-based probe serves as proof-of-principle for the use of these shell protein functionalizations for assessing shell permeability to small molecules in real time. In principle, any encapsulated enzymatic transformation(s) that results in a spectroscopic signal could serve as a readout of shell permeability, enabling the characterization of shell permeability to physiologically relevant species such as NAD(P)H. Leveraging the plasticity of subunit composition combined with altered selectivity from pore mutagenesis could allow for the facile creation of shells with bespoke permeabilities—likely a vital step toward increasing efficiencies of encapsulated, non-native pathways. The development of the EnCo and CAP methods represent significant steps towards more accessible physiological studies as well as realizing the biotechnological promises of bacterial microcompartments.

## Methods

### Shell expression

BL21(DE3) strains harboring shell plasmids were grown to OD600 0.6–0.8 in lysogeny broth at 37 °C with 100 µg/mL ampicillin and induced with 50 µM IPTG with a brief cold-shock on ice. When co-expressing SpyTag/SpyCatcher cargo or P_SII_, strains were additionally cultured with 50 µg/mL kanamycin and induced simultaneously with 5 ng/mL anhydrotetracycline. Incubation was then continued at 18 °C for 16–20 h.

### Classical shell purifications

Shells were prepared using methods described in ref. ^2^. Briefly, the cell pellet from a 2 L culture expressing pHT_1_T_2_T_3_ was resuspended in Tris-buffered saline (20 mM Tris, 50 mM NaCl, pH 7.4 TBS 20/50 hereafter), lysed by French press and clarified by centrifugation at 25,000×*g* for 30 min. After sucrose cushion and sucrose gradient centrifugations, shells were further purified by application to a MonoQ 10/100 GL anion-exchange column connected to an Äkta Pure system and elution with a shallow sodium chloride gradient (200–400 mM NaCl over 10 column volumes). Shell containing fractions were pooled and concentrated with a 15 mL 100 kDa MWCO filter (Amicon) and stored on ice after the addition of 0.02% sodium azide as a preservative. For HT_SC~ST_probeT_2_T_3_ shells, classically purified HT_SC_T_2_T_3_ shells were incubated overnight with an excess of purified _ST_probe (see Supplementary Methods/Expression and purification of _ST_probe protein) at 4 °C and then excess _ST_probe was removed via application to a HisTrap column, followed by another MonoQ step. All buffers for the HT_SC~ST_probeT_2_T_3_ shell preparation were amended with 5 mM EDTA and 5 mM TCEP to maintain reducing conditions.

### Complementation-based affinity purification

For initial in vivo and ex vivo capping, cell pellets from 1 L pHT_1_T_2_T_3_ cultures were lysed using BPER-II amended with recombinant lysozyme and benzonase according to manufacturer’s recommendation and clarified by centrifugation as above. Shell lysates were then mixed with clarified P_SII_ lysates (2:1 v/v ratio of shell to pentamer pellets for HT_1_T_2_T_3_ shells; 10:1 for all minimal shells; see Supplementary Methods/Expression and purification of P_SII_ protein) and incubated for 30 min to allow capping to occur. Lysates were then applied to a 5 mL StrepTrap (GE Healthcare) column equilibrated with Buffer A (100 mM Tris-HCl, pH 7.4, 150 mM NaCl, 1 mM EDTA). The column was washed with six column volumes Buffer A and proteins were eluted in six column volumes Buffer B (20 mM Tris-HCl, pH 7.4, 50 mM NaCl, 1 mM EDTA, 2.5 mM *D*-desthiobiotin). Where specified, shells were further purified with anion-exchange chromatography and/or concentrated with 100 kDa molecular weight cut-off Amicon spin filters as described. Capping experiments involving encapsulated cargo (Figs. 4, 5) were scaled down to 0.2 L shell cultures and purified with 1 mL StrepTrap columns using otherwise identical methods.

### Pentamer titration

Ex vivo capping was performed as described above except a range of clarified P_SII_ lysate amounts (25, 10, 2.5, 1.25, and 0 mL equivalents) was added to 0.2 L equivalents pHT_1_T_2_T_3_ shell lysate.

### Ex vivo loading of cargo into minimal shells

Cultures of pET11n::HT_SC/ST_, pBbA2k::_ST/SC_mTurquoise2-6xHis and pBbA2k::_ST_SYFP2-6xHis were induced with 50 µM IPTG (pET11n::HT_SC/ST_) or 50 ng/mL aTc (pBbA2k::_ST/SC_mTurquoise2-6xHis, pBbA2k::_ST_SYFP2-6xHis). Biological replicates (total *n* = 2) were performed with freshly re-transformed strains. Cell pellets were resuspended in Buffer A (4 mL per gram cell paste) supplemented with 12 µl benzonase (25 U/µl), a pinch of hen egg lysozyme and one Roche Complete EDTA-free protease inhibitor tablet, lysed using a French press and clarified by centrifugation as described. For co-encapsulation experiments, 0.2 L equivalents of the HT_SC_ expressing lysate was mixed with a total of 0.2 L equivalent _ST_cfp/_ST_yfp lysates at varying ratios where one part corresponds to 20 mL equivalents lysate. For encapsulation of a single species (control reactions and loading into HT_ST_ shells), 0.2 L equiv. of shell lysate was mixed with 0.2 L equiv. cargo. Mixtures were incubated at room temperature overnight and then incubated with clarified P_SII_ lysate and purified via 1 mL StrepTrap columns as described and concentrated with 0.5 mL 100 kDa MWCO spin filters to obtain sufficient fluorescent signal. For fluorescence comparisons to encapsulated _ST_cfp/_ST_yfp, the flow-through during sample application to the StrepTrap columns was collected.

### Permeability experiments with FlAsH-EDT_2_ and TEVp

HT_SC_~_ST_probeT_2_T_3_ preparations (final protein concentration of ~1 mg/mL) or unencapsulated _ST_probe (~100 µg/mL) in 50 mM Tris-HCl, pH 7.4, 150 mM NaCl, 5 mM EDTA, 5 mM TCEP, 1 mM β-mercaptoethanol were incubated in the absence or presence of excess P_SII_ in microtiter plates for sixty minutes. A FlAsH-EDT_2_ and 2,3-dimercapto-1-propanol solution (1 mM and 9 mM, respectively, in DMSO) was added such that the final FlAsH-EDT_2_ concentration was 10 µM and incubated at room temperature for 2 h. TEV protease was then added to a final concentration of 40 µg/mL (~1:20 w/w) and incubated for 2 h.

Refer to Supplementary Methods for additional notes on the following topics: chemicals and reagents; SDS-PAGE and western blot analysis of protein preparations; fluorescence intensity, spectra readings, and fluorescence normalization; electron microscopy. Plasmid construction is summarized in Supplementary Table 1. Full-frame TEM micrographs were provided in Supplementary Fig. 10.

### Data availability

Data supporting the findings of this study are available from the corresponding author upon reasonable request.

## Electronic supplementary material

Supplementary Information
