# Supplementary material for: Programmed loading and rapid purification of engineered bacterial microcompartment shells
Source: Nat Commun. 2018 Jul 23;9:2881. doi: 10.1038/s41467-018-05162-z (PMC6056538; doi:10.1038/s41467-018-05162-z)
Supplement: Supplementary file 1 — Supplementary Information [file 41467_2018_5162_MOESM1_ESM.docx]

**Programmed loading and rapid purification of engineered bacterial microcompartment shells**

Hagen *et al.*

**Supplementary Methods**

**Chemicals and Reagents**

FlAsH-EDT_2_ was purchased from Carbosynth (Compton, United Kingdom), all other chemicals were purchased from Millipore-Sigma (St. Louis, USA).

**SDS-PAGE and Western blot analysis of protein preparations**

Shell preparations were typically normalized to A280 = 1, denatured in reducing sample buffer and loaded on 4–20% Mini-PROTEAN® TGX™ Precast Protein Gels (Bio-Rad, USA). When comparing shell yields (e.g. pentamer titration experiment), samples were loaded with equal volumes rather than being normalized to A280. Gels were washed and stained with SimplyBlue™ SafeStain (Thermo Fisher, USA). Western blotting was performed by electrotransfer of proteins to nitrocellulose membranes which were then blocked with phosphate-buffered saline (PBS) + 5% (w/v) non-fat dry milk, 0.1% (v/v) Triton X-100. A 1:10,000 dilution of 6x-His Tag Horseradish Peroxidase antibody (ThermoFisher cat# MA1-21315-HRP) in the above buffer was then applied to the membrane and after one hour, washed extensively with the above buffer. A final wash with PBS + 0.1% Triton X-100 was performed and the blot was developed using SuperSignal West Pico Chemiluminescent Substrate (ThermoFisher cat# 34087) according to manufacturer’s recommendations. Gels and western blots were imaged with ChemiDoc™ XRS+ System and analytical densitometry performed with Image Lab™ Software (Bio-Rad).

**Expression and purification of P_SII_ protein**

BL21(DE3)/ pBbE2k::P_SII_ strain was grown to OD600 0.6-0.8 at 37°C, induced with 50 ng/mL anhydrotetracycline (aTc) and harvested after an additional 4-6 h incubation at 37°C. Pellets were lysed with BPER-II as described. Insoluble debris was cleared via centrifugation and the supernatant applied to 5 mL StrepTrap (GE Healthcare) column equilibrated with Buffer A. The column was washed with 30 mL Buffer A and proteins were eluted in Buffer A supplemented with 2.5 mM *D*-desthiobiotin. Eluate was concentrated with 30 kDa molecular weight cut-off filters (Amicon) and further purified and buffer exchanged into 20 mM Tris-HCl pH 7.4, 50 mM NaCl via size-exclusion chromatography (HiLoad 16/600 Superdex 75 prep grade, GE Healthcare).

**Expression and purification of _ST_probe protein**

BL21(DE3)/pProbe strain was cultured and induced as with the P_SII_ preparation. Cells were lysed by French press and 20 mM imidazole and 2.5 mM TCEP was added to clarified lysate and applied to a 5 mL HisTrap column (GE Healthcare) equilibrated with Buffer C (20 mM Tris-HCl pH 7.4, 300 mM NaCl, 20 mM imidazole, 2.5 mM TCEP). Column was washed with 10 column volumes Buffer C and eluted in a small volume with Buffer D (20 mM Tris-HCl pH 7.4, 300 mM NaCl, 100 mM imidazole, 2.5 mM TCEP). 5 mM EDTA, and 5 mM additional TCEP were added to eluate which was then further purified and buffer exchanged into 20 mM Tris-HCl pH 7.4, 50 mM NaCl, 5 mM EDTA, 5 mM TCEP via size-exclusion chromatography (HiLoad 16/600 Superdex 75 prep grade, GE Healthcare).

**TEV protease purification**

TEV protease was purified using the protocol described in^1^.

**Fluorescence intensity, spectra readings and fluorescence normalization**

An M1000 or Spark plate reader (Tecan, Switzerland) was used to collect all fluorescence measurements in 96-well microplates with 50-100 µl of sample per well, in top-mode. Fluorescence intensity readings were collected with excitation and emission bandwidths of 5 nm; 434/474 nm for CFP and 515/530 nm for YFP. Emission spectra scans were collected via excitation at 405 nm and emission collection from 450 to 600 nm using 5 nm steps. For each individual experiment and fluorophore, the gain was optimized automatically to the well with the highest fluorescence value to avoid saturating the detector. For the _ST_cfp/_ST_yfp *ex vivo* loading experiment, the fluorescence intensity values for CFP only and YFP only (10:0 and 0:10, respectively) samples were normalized to 100 and all other values scaled according to the same factor. Emission spectra scans were normalized to total fluorescence emission (i.e. area under the curve of the emission scan from 430-650 nm) as the total fluorescence signal varied widely due to the differing amounts of FRET donor/acceptor present in each sample.

**Molecular modeling and images**

UCSF Chimera^2^ and PyMol (The PyMOL Molecular Graphics System, Version 2.0 Schrödinger, LLC.) were used for molecular visualizations and figure creation. To create the T_SC_ model, the original atomic coordinates of HO BMC-T1 (PDB: 5DIH) were retrieved and the “GASGA” linker was deleted to split the chain into two fragments. The poly-G/S linker was made de novo using Chimera’s build function and concatenated to BMC-T_1_ fragment 1. The SpyCatcher atomic coordinates (PDB: 4MLI) were retrieved and trimmed appropriately to make the ΔN1ΔC2 variant which was then concatenated to the poly-G/S linker. The second poly-G/S linker was made as before and appended to the SpyCatcher domain. Bonds in the poly-G/S linkers were manually torsioned to juxtapose the C-terminus of the second poly-G/S linker to the N-terminus of the second BMC-T_1_ fragment and a peptide bond created between the two. The T_ST_ model was made by replacing the SpyCatcher domain in the T_SC_ model with a de novo generated SpyTag modeled as a beta-strand. The structures were imported into FoldIt^3^ and allowed to minimize through side-chain and backbone “wiggling” with some manual guidance.

**Electron Microscopy**

For TEM analysis, 6 µl of purified shells at an A280 between 0.1-1 were mounted on formvar/carbon-coated copper grids (Electron Microscopy Sciences, No. 456 FCF300-Cu) for 30 seconds and then wicked away with filter paper. The grids were washed three times in 6 µl drops of water, dried with filter paper and then negatively stained with 5 µl 1% (w/v) aqueous uranyl acetate except for Halo shells which were stained with 1% (w/v) ammonium molybdate. After ten seconds, the stain was wicked off and the grids allowed to dry. Images were taken on a Tecnai 12 TEM operated at an accelerating voltage of 120 kV using an Ultrascan 1000 2k x 2k CCD camera.

**
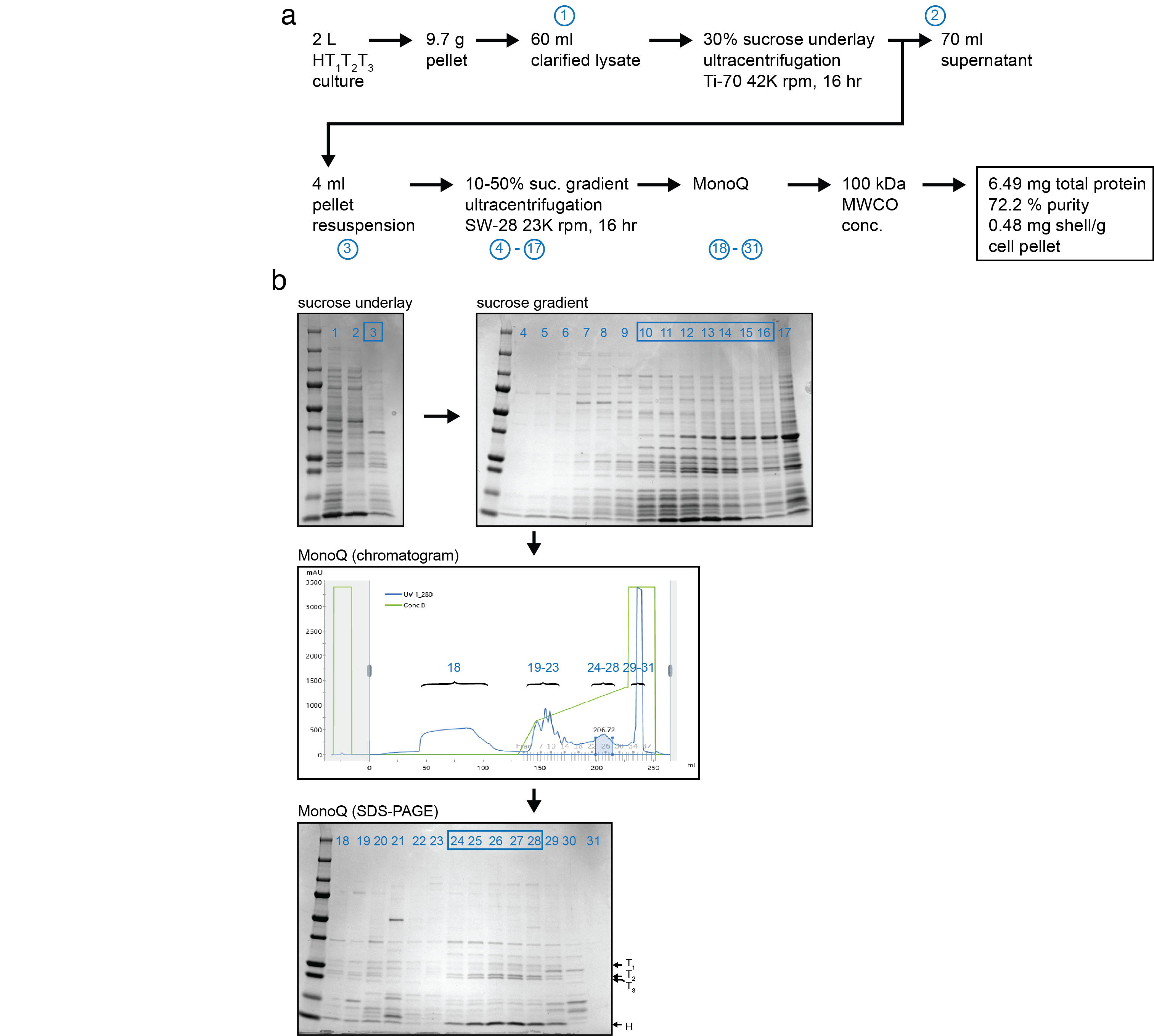
**

**Supplementary Figure 1. Purification scheme and purification tracking of HT_1_T_2_T_3_ classic shell preparation**

a. Purification scheme, purity and yield for the aforementioned shells. Circled numbers correspond to samples analyzed by SDS-PAGE in part (b)

b. Purification tracking of the aforementioned shells including SDS-PAGE and chromatographic analysis. Boxed numbers correspond to shell-containing fractions that were saved for subsequent processing.

**
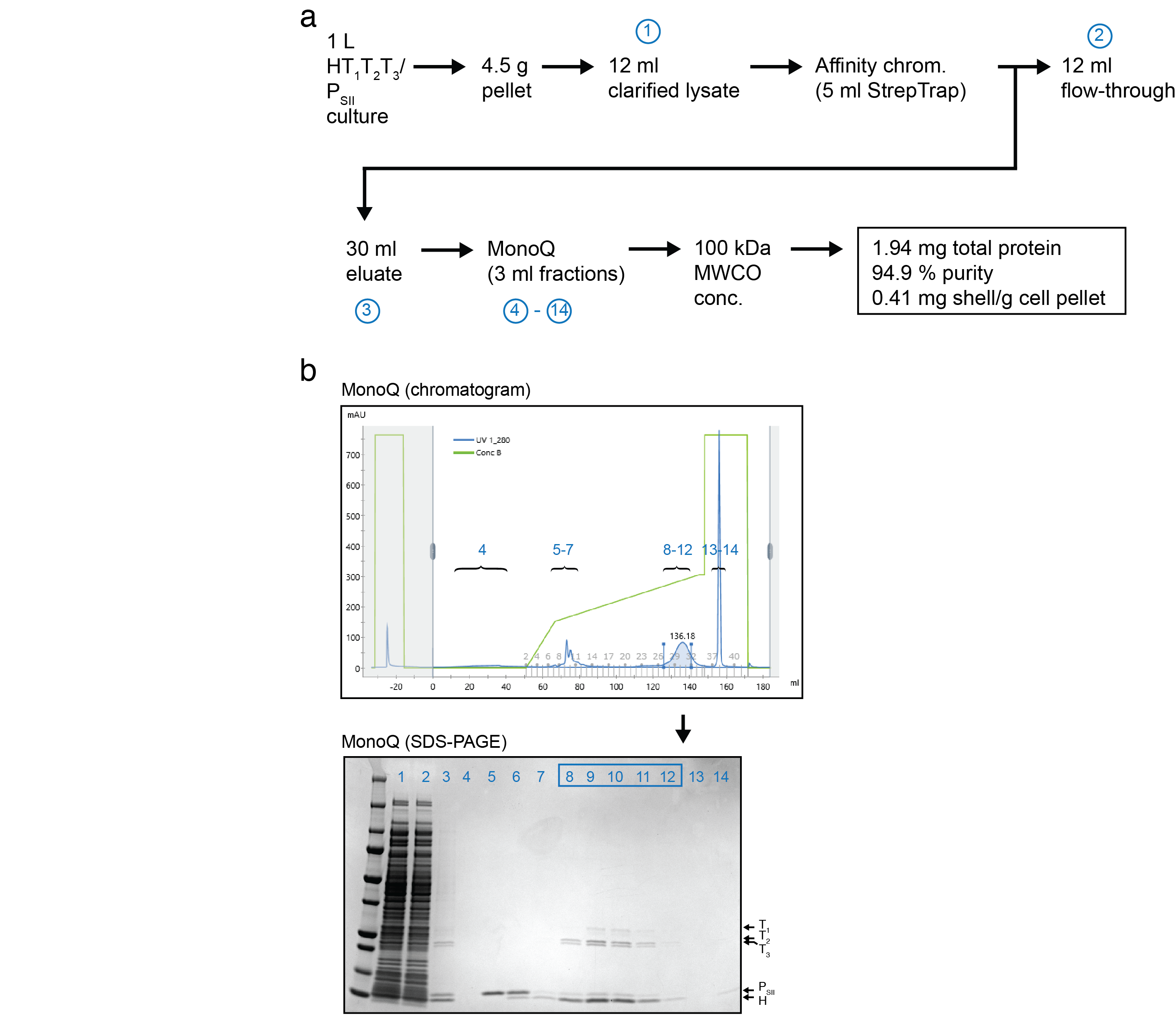
**

**Supplementary Figure 2. Purification scheme and purification tracking of HT_1_T_2_T_3_P_SII_ *in vivo* CAP shell preparation**

a. Purification scheme, purity and yield for the aforementioned shells. Circled numbers correspond to samples analyzed by SDS-PAGE in part (b)

b. Purification tracking of the aforementioned shells including SDS-PAGE and chromatographic analysis. Boxed numbers correspond to shell-containing fractions that were saved for subsequent processing.

**
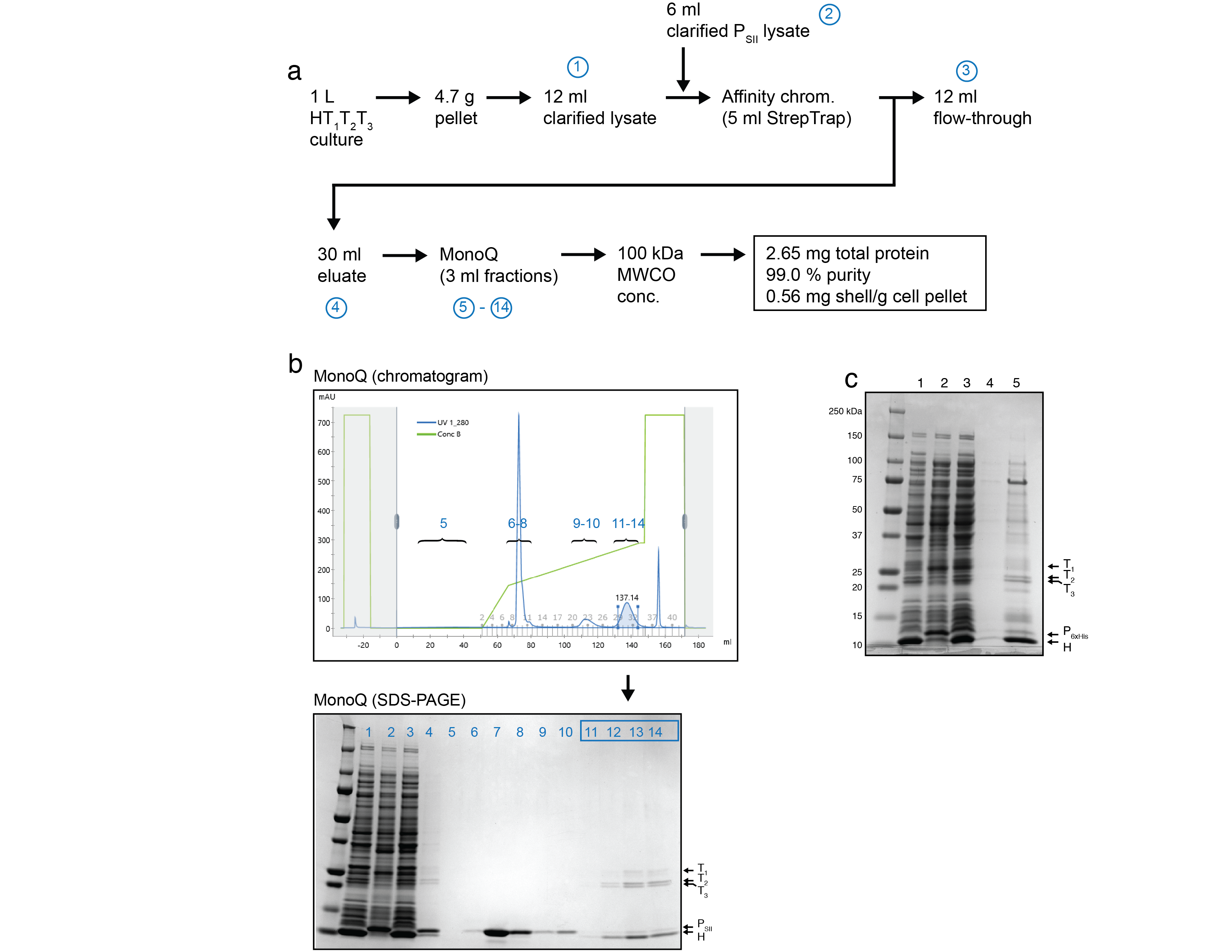
**

**Suplementary Figure 3. Purification scheme and purification tracking of HT_1_T_2_T_3_P_SII_ *ex vivo* CAP shell preparation and control purification with non-SII tagged pentamer**

a. Purification scheme, purity and yield for the aforementioned shells. Circled numbers correspond to samples analyzed by SDS-PAGE in part (b)

b. Purification tracking of the aforementioned shells including SDS-PAGE and chromatographic analysis. Boxed numbers correspond to shell-containing fractions that were saved for subsequent processing.

c. SDS-PAGE analysis of an *ex vivo* CAP shell preparation using hexahistidine-tagged pentamers. Lane 1-2: clarified lysates of HT_1_T_2_T_3_ and P_6xHis_ cultures. Lane 3: StrepTrap flow through Lane 4: Neat StrepTrap eluate. Lane 5: StrepTrap eluate concentrated 50-fold

**
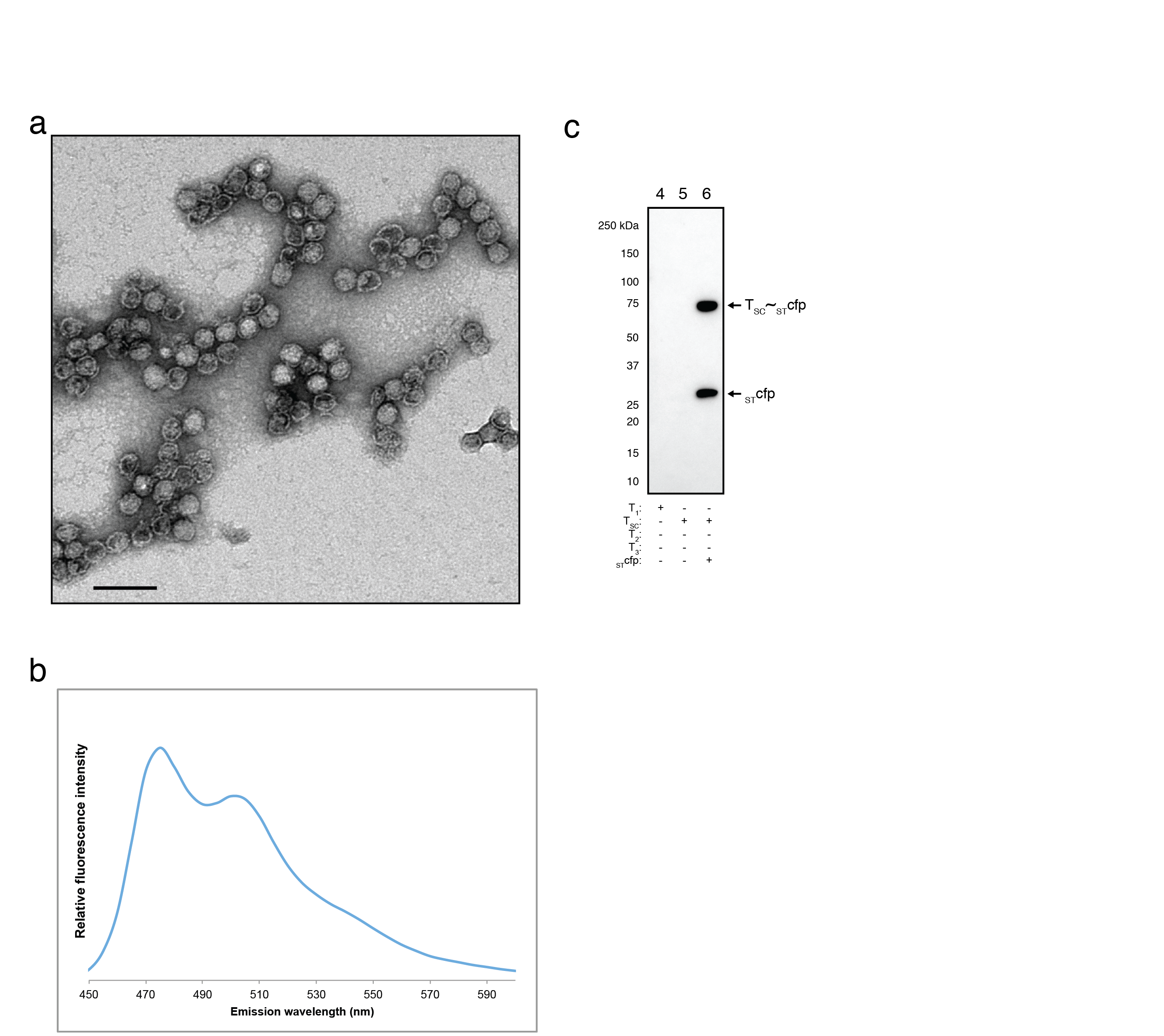
**

**Supplementary Figure 4. Characterization of various shell preparations**

a. TEM analysis of HT_SC_~_ST_cfpT_2_T_3_P_SII_ shells revealing normal morphology. Scale bar = 100 nm

b. Representative emission spectrum of _ST_cfp-loaded shells

c. anti-His western blot of a subset of shell preparations. Composition of shell preparations given in tabular form below each lane; lane numbers correspond to those in Figure 4a

**
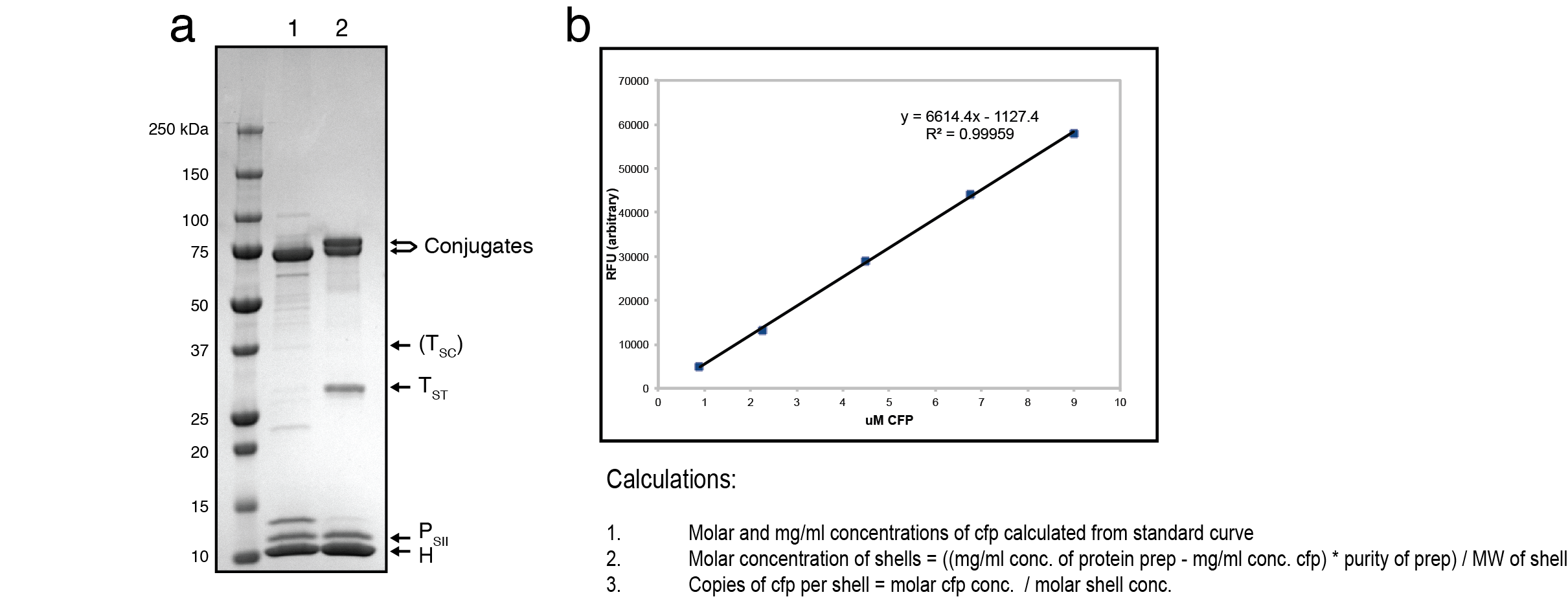
**

**Supplementary Figure 5. Analysis of yield, purity and encapsulation efficiency of both EnCo architectures**

a. SDS-PAGE analysis of final shell preparations. Lane 1: HT_SC_~_ST_cfpP_SII_ shells, Lane 2: HT_ST_~_SC_cfpP_SII_ shells

b. Standard curve of fluorescence units with respect to cfp concentration and calculations used for determining cfp copies per shell


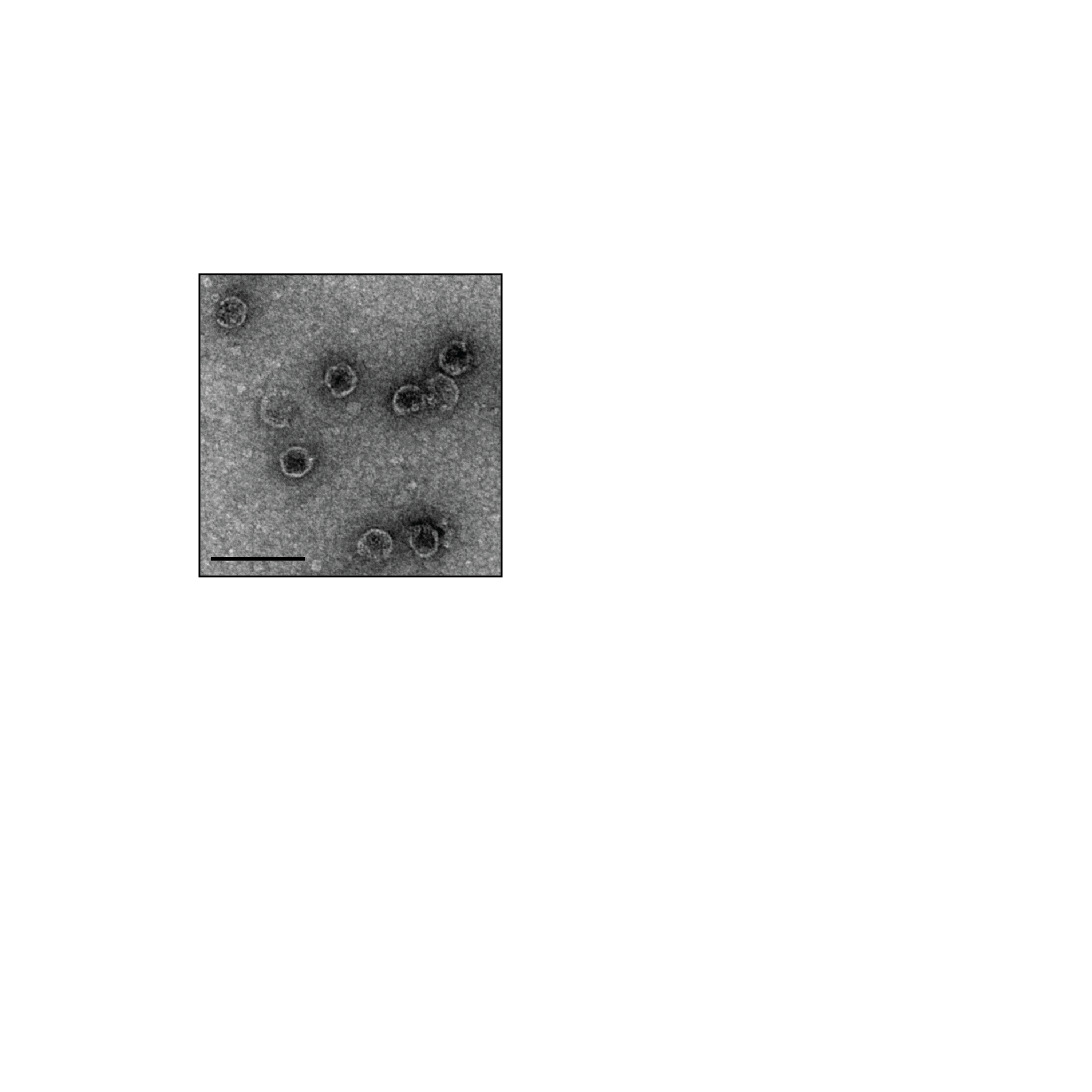


**Supplementary Figure 6. Negative stain TEM micrograph of unloaded HT_SC_P_SII_ shell preparations**

Scale bar = 100 nm

**
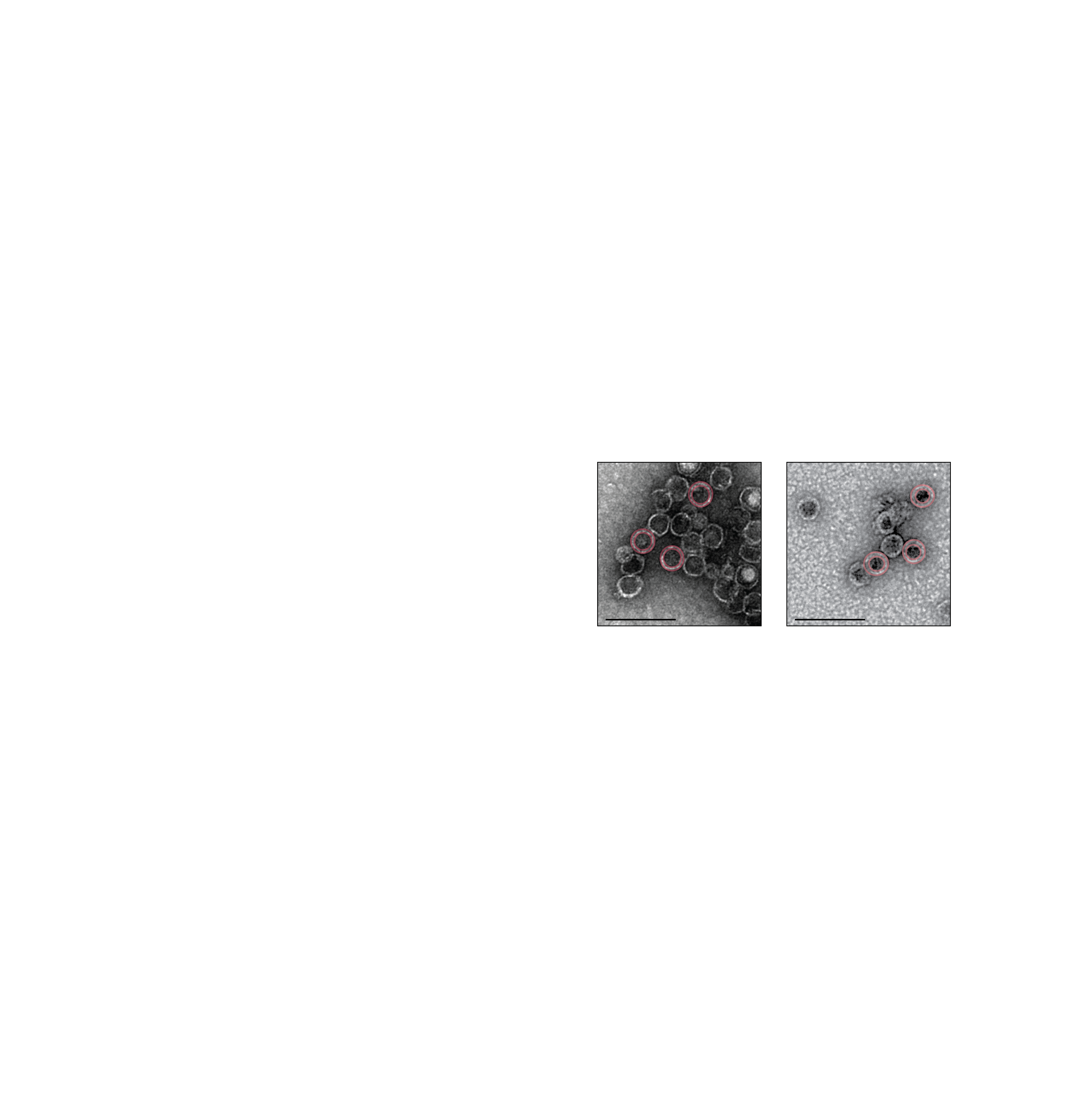
**

**Supplementary Figure 7. Shell thickness measurements of HT_1_P_SII_ and HT_SC_~_ST_cfpP_SII_ shell**

Left panel: HT_1_P_SII_ shells. RIght panel: HT_SC_~_ST_cfpP_SII_ shells. Inner and outer edges of shells are circled and thickness is calculated using the formula: (outer diameter - inner diameter)/2

Scale bars = 100 nm

**
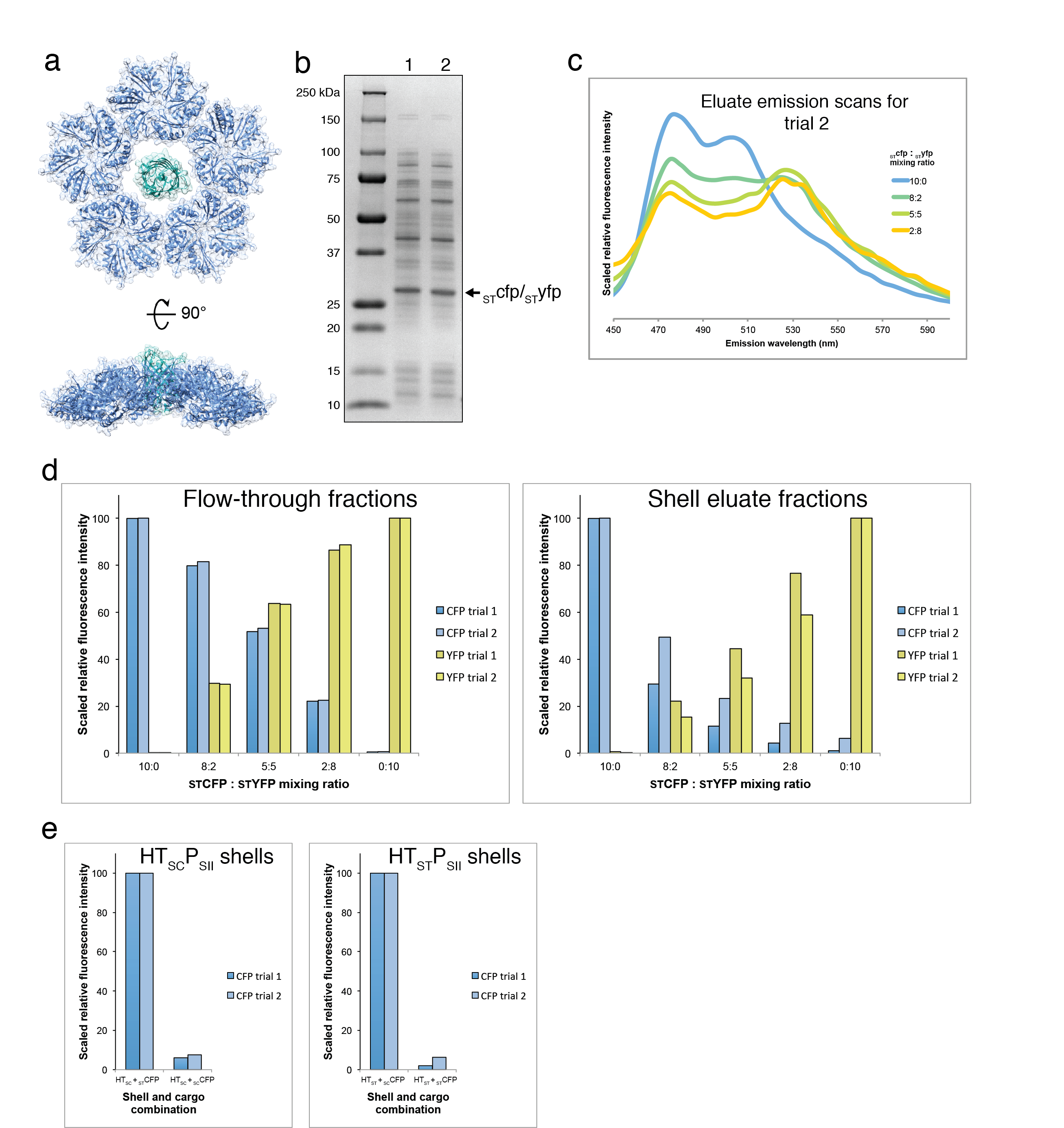
**

**Supplementary Figure 8. Co-encapsulation experiment modelling, SDS-PAGE analysis and fluorometry data**

a. Model derived from Sutter 2017 depicting five hexamer subunits that abut HO shell pentamers with mTurquoise2 placed in pentamer void. Top: View from outside of the shell. Bottom: Side view

b. SDS-PAGE analysis of whole cell lysates. Lane 1: _ST_cfp, Lane 2: _ST_yfp

c. Scaled emission spectra (excitation: 405 nm) of programmed cargo, trial 2

d. Left panel: Relative fluorescence intensities of flow-through fractions (unencapsulated fluorophores)

Right panel: Relative fluorescence intensities of shell fractions (encapsulated fluorophores)

Note attenuated emission of _ST_cfp when encapsulated relative to unencapsulated _ST_cfp

e. Relative fluorescence intensities of shell eluates comparing cognate and non-cognate cargo loading. Left panel: HT_SC_P_SII_ shells. Right panel: HT_ST_P_SII_ shells

**
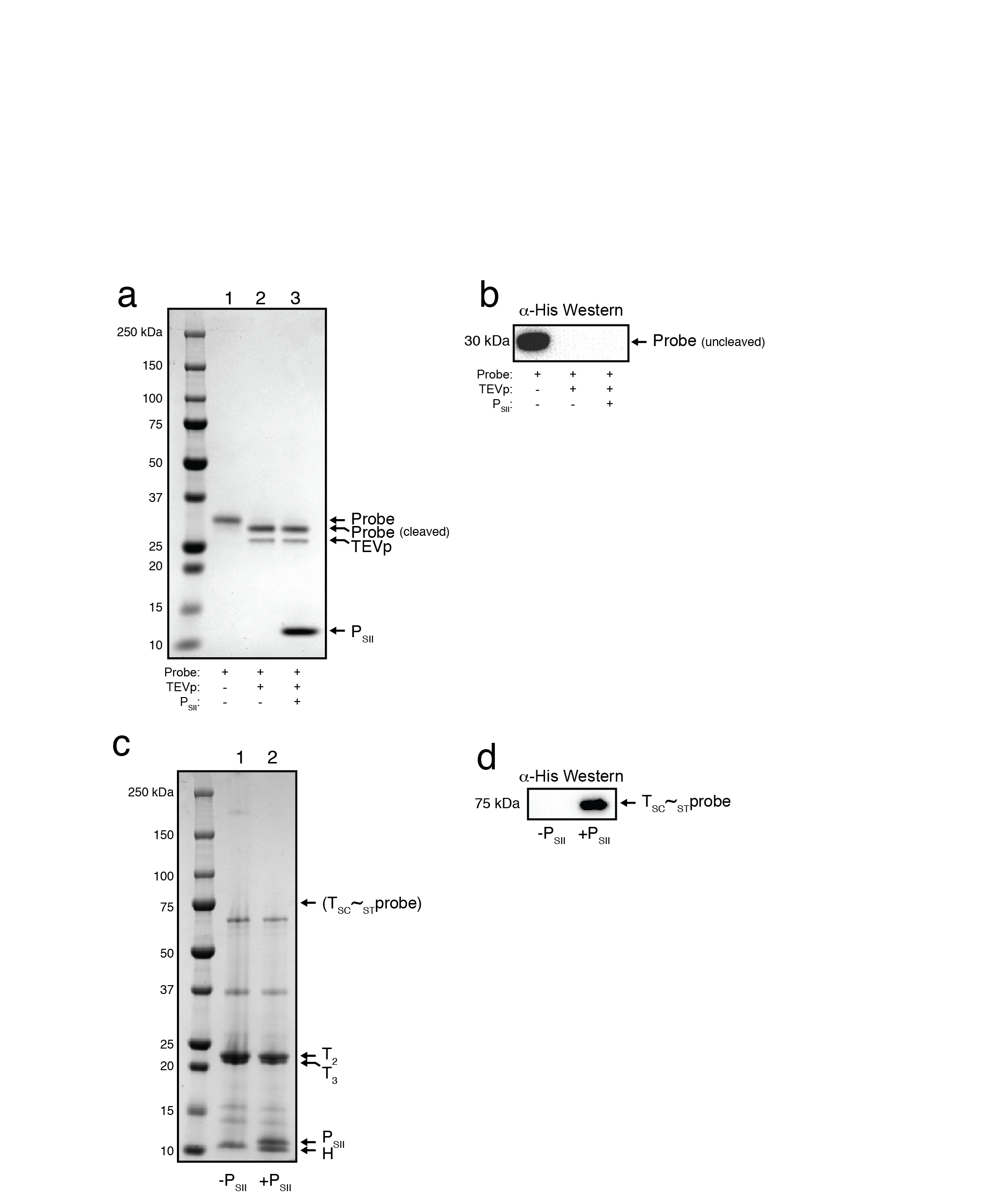
**

**Supplementary Figure 9. SDS-PAGE analysis and western blot of unencapsulated probe and samples at conclusion of permeability experiment**

a. SDS-PAGE analysis of unencapsulated probe in presence of TEVp and P_SII_. The C-terminal cleavage product is approximately 2.0 kDa and too small for detection

b. anti-His western blot of samples in (a)

c. SDS-PAGE of uncapped shells (lane 1) and capped shells (lane 2)

d. anti-His western blot of samples in (c)

**
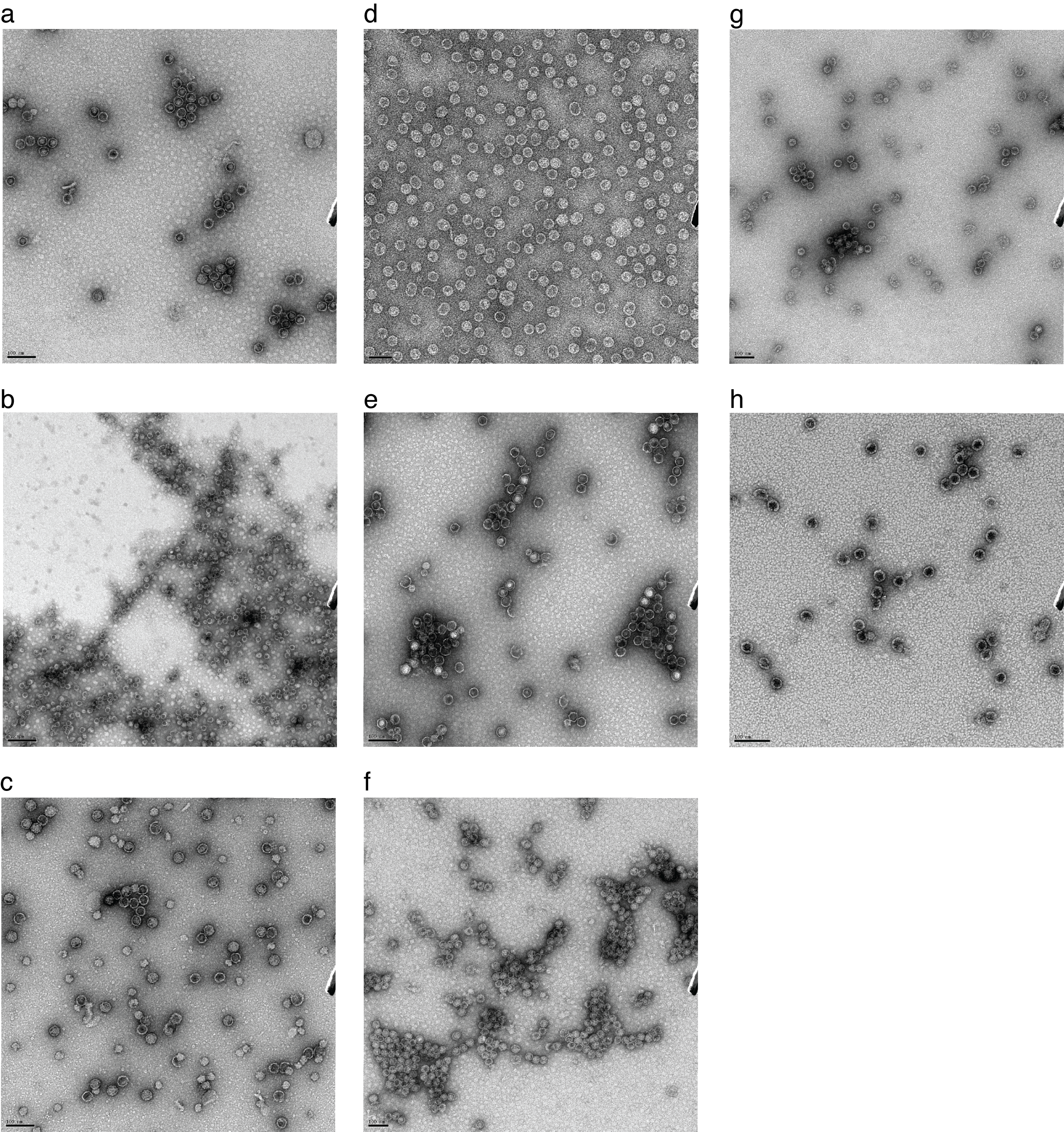
**

**Supplementary Figure 10. Full-frame TEM micrographs corresponding to indicated figures**

a. Corresponds to figure 1a. Scale bar = 100 nm

b. Corresponds to figure 1b. Scale bar = 200 nm

c. Corresponds to figure 1c. Scale bar = 100 nm

d. Corresponds to figure 1g. Scale bar = 50 nm

e. Corresponds to figure 2b. Scale bar = 100 nm

f. Corresponds to figure 2c. Scale bar = 100 nm

g. Corresponds to figure 2d. Scale bar = 100 nm

h. Corresponds to figure 4c. Scale bar = 100 nm

**Supplementary Table 1. Details of plasmids used in current study**

| Alias | Plasmid identity | Nucleotide sequence of open-reading frame (engineered proteins only) | Construction Notes |
| --- | --- | --- | --- |
| pHT_1_T_2_T_3_ | pET11n::HT_1_T_2_T_3_ |  | Described in Sutter et al 2017^4^. In brief, the HO hexamer and three trimers are driven polycistronically from an inducible T7 promoter. A strong ribosome-binding site (RBS) precedes the hexamer and medium RBSs precede the trimers. |
| pARH292 | pBbE2k::P_SII_ | ATGGTCCTGGGTAAAGTCGTGGGTACGGTGGTGGCGAGCCGCAAAGAACCGCGCATTGAAGGTCTGAGCCTGCTGCTGGTCCGTGCCTGCGATCCGGACGGTACCCCGACGGGTGGTGCAGTGGTTTGTGCAGATGCAGTGGGTGCAGGTGTTGGTGAAGTCGTGCTGTATGCGAGTGGCAGCTCTGCCCGTCAGACCGAAGTCACGAACAATCGCCCGGTTGATGCAACCATTATGGCTATCGTTGACCTGGTCGAAATGGGCGGTGATGTGCGTTTTCGCAAAGACGGCAGCTGGAGTCATCCTCAGTTTGAAAAATAA | Pentamer Hoch5814 was PCR amplified from the *Haliangium ochraceum* synthetic operon^5^ to include a C-terminal Strep-II tag and cloned into pBbE2k^6^ |
| pARH329 | pET11n::HT_1_ |  | Inverse PCR was used to delete trimers T_2_ and T_3_ (Hoch5816 and Hoch3341, respectively) from pHT_1_T_2_T_3_ |
| pARH317 | pET11n::HT_2_ |  | Inverse PCR was used to delete trimers T_1_ and T_3_ (Hoch5812 and Hoch3341, respectively) from pHT_1_T_2_T_3_ |
| pARH387 | pET11n::HT_3_ |  | Gibson assembly was used to remove trimers T_1_ and T_2_ (Hoch5812 and Hoch5816, respectively) from pHT_1_T_2_T_3_ |
| pARH346 | pET11n::HT_ST_ | ATGGACCACGCTCCGGAACGCTTTGATGCGACCCCGCCGGCAGGTGAACCGGACCGCCCGGCACTGGGTGTGCTGGAACTGACCTCAATTGCTCGTGGTATCACCGTTGCGGATGCGGCCCTGAAACGTGCACCGAGTCTGCTGCTGATGTCCCGCCCGGTCAGCTCTGGCAAGCATCTGCTGATGATGCGTGGCCAGGTGGCAGAAGTTGAAGAATCAATGATTGCAGCTCGCGAAATCGCTGGTGCAGGTGGTGGATCAGGTGGTAGTGCTCATATTGTCATGGTTGATGCTTACAAGCCAACTAAGGGAGGTAGCGGAGGAAGTGGTGCTCTGCTGGATGAACTGGAACTGCCGTATGCGCACGAACAACTGTGGCGCTTTCTGGACGCACCGGTGGTTGCAGATGCATGGGAAGAAGACACCGAAAGCGTCATTATCGTGGAAACCGCGACGGTGTGCGCGGCCATTGATAGTGCCGACGCAGCTCTGAAAACGGCACCGGTCGTGCTGCGTGATATGCGCCTGGCCATTGGTATCGCTGGCAAGGCGTTTTTCACCCTGACGGGTGAACTGGCAGACGTGGAAGCGGCCGCAGAAGTTGTCCGTGAACGTTGCGGTGCACGTCTGCTGGAACTGGCATGTATCGCACGCCCGGTTGATGAACTGCGTGGCCGCCTGTTTTTCTAA | Inverse PCR was used to introduce the SpyTag sequence flanked by poly-Gly/Ser linkers into the T_1_ (Hoch5812) domain of plasmid pARH329 after amino acid Gly84 (...AGAG[insertion]SGA...) |
| pARH349 | pET11n::HT_ST_T_2_T_3_ |  | The same cloning strategy employed to create pARH346 was employed using pHT_1_T_2_T_3_ as template |
| pARH353 | pET11n::HT_SC_ | ATGGACCACGCTCCGGAACGCTTTGATGCGACCCCGCCGGCAGGTGAACCGGACCGCCCGGCACTGGGTGTGCTGGAACTGACCTCAATTGCTCGTGGTATCACCGTTGCGGATGCGGCCCTGAAACGTGCACCGAGTCTGCTGCTGATGTCCCGCCCGGTCAGCTCTGGCAAGCATCTGCTGATGATGCGTGGCCAGGTGGCAGAAGTTGAAGAATCAATGATTGCAGCTCGCGAAATCGCTGGTgcaggtGGTGGATCAGGTGGTAGTGATAGTGCTACCCATATTAAATTCTCAAAACGTGATGAGGACGGCAAAGAGTTAGCTGGTGCAACTATGGAGTTGCGTGATTCATCTGGTAAAACTATTAGTACATGGATTTCAGATGGACAAGTGAAAGATTTCTACCTGTATCCAGGAAAATATACATTTGTCGAAACCGCAGCACCAGACGGTTATGAGGTAGCAACTGCTATTACCTTTACAGTTAATGAGCAAGGTCAGGTTACTGTAAATGGAGGTAGCGGAGGAAGTggtgctCTGCTGGATGAACTGGAACTGCCGTATGCGCACGAACAACTGTGGCGCTTTCTGGACGCACCGGTGGTTGCAGATGCATGGGAAGAAGACACCGAAAGCGTCATTATCGTGGAAACCGCGACGGTGTGCGCGGCCATTGATAGTGCCGACGCAGCTCTGAAAACGGCACCGGTCGTGCTGCGTGATATGCGCCTGGCCATTGGTATCGCTGGCAAGGCGTTTTTCACCCTGACGGGTGAACTGGCAGACGTGGAAGCGGCCGCAGAAGTTGTCCGTGAACGTTGCGGTGCACGTCTGCTGGAACTGGCATGTATCGCACGCCCGGTTGATGAACTGCGTGGCCGCCTGTTTTTCTAA | Gibson assembly^7^ was used to replace the SpyTag region in pARH346 with the SpyCatcher∆N1∆C2 variant domain^8^ while maintaining the insulating poly-Gly/Ser linkers. |
| pARH355 | pET11n::HT_SC_T_2_T_3_ |  | The same cloning strategy employed to create pARH353 was employed using pARH349 as template |
| pARH360 | pBbA2k::6xHis-_SC_mTurquoise2 | ATGTCGTACTACCATCACCATCACCATCACGATTACGACATCCCAACGACCGAAAACCTGTATTTTCAGGGCGCCATGGTTGATACCTTATCAGGTTTATCAAGTGAGCAAGGTCAGTCCGGTGATATGACAATTGAAGAAGATAGTGCTACCCATATTAAATTCTCAAAACGTGATGAGGACGGCAAAGAGTTAGCTGGTGCAACTATGGAGTTGCGTGATTCATCTGGTAAAACTATTAGTACATGGATTTCAGATGGACAAGTGAAAGATTTCTACCTGTATCCAGGAAAATATACATTTGTCGAAACCGCAGCACCAGACGGTTATGAGGTAGCAACTGCTATTACCTTTACAGTTAATGAGCAAGGTCAGGTTACTGTAAATGGCAAAGCAACTAAAGGTGACGCTCATATTGGTGGAGGTTCAGGCGGTGCTAGCGTTAGCAAAGGCGAAGAACTGTTCACCGGTGTAGTGCCGATCCTGGTTGAGCTGGATGGCGACGTGAATGGCCACAAATTCAGCGTGTCCGGTGAAGGCGAAGGTGATGCCACGTATGGTAAGCTGACCCTGAAATTTATCTGCACCACTGGTAAGCTGCCGGTTCCGTGGCCGACGCTGGTTACGACCCTGTCCTGGGGCGTGCAGTGTTTCGCCCGCTATCCGGACCACATGAAACAGCATGACTTCTTCAAAAGCGCAATGCCGGAGGGTTACGTGCAGGAACGTACCATCTTTTTCAAGGACGACGGTAACTACAAGACTCGTGCAGAAGTGAAATTTGAAGGCGATACCCTGGTTAACCGTATTGAACTGAAAGGTATCGATTTCAAAGAAGACGGCAACATCCTGGGCCACAAACTGGAATACAATTATTTTAGCGATAACGTATACATCACCGCAGACAAACAGAAAAACGGCATTAAAGCAAACTTCAAAATCCGTCACAACATTGAAGACGGTGGCGTCCAGCTGGCAGACCATTACCAGCAGAACACCCCGATTGGTGACGGCCCGGTTCTGCTGCCGGACAACCACTATCTGTCCACTCAGAGCAAACTGTCCAAAGATCCGAACGAGAAACGCGACCACATGGTGCTGCTGGAATTTGTTACCGCTGCTGGCATCACTCTGGGTATGGATGAGCTGTACAAATAA | Gibson assembly was used to introduce the 6xHis-SpyCatcher and an *E. coli* codon-optimized version of mTurquoise2 (Integrated DNA Technologies, Coralville USA;^9^) domains into pBbA2k^6^ |
| pARH364 | pBbA2k::_ST_mTurquoise2-6xHis | ATGGGCGAGTTGGCTCACATTGTTATGGTTGACGCATATAAGCCGACAAAGGGAAGTGTTAGCAAAGGCGAAGAACTGTTCACCGGTGTAGTGCCGATCCTGGTTGAGCTGGATGGCGACGTGAATGGCCACAAATTCAGCGTGTCCGGTGAAGGCGAAGGTGATGCCACGTATGGTAAGCTGACCCTGAAATTTATCTGCACCACTGGTAAGCTGCCGGTTCCGTGGCCGACGCTGGTTACGACCCTGTCCTGGGGCGTGCAGTGTTTCGCCCGCTATCCGGACCACATGAAACAGCATGACTTCTTCAAAAGCGCAATGCCGGAGGGTTACGTGCAGGAACGTACCATCTTTTTCAAGGACGACGGTAACTACAAGACTCGTGCAGAAGTGAAATTTGAAGGCGATACCCTGGTTAACCGTATTGAACTGAAAGGTATCGATTTCAAAGAAGACGGCAACATCCTGGGCCACAAACTGGAATACAATTATTTTAGCGATAACGTATACATCACCGCAGACAAACAGAAAAACGGCATTAAAGCAAACTTCAAAATCCGTCACAACATTGAAGACGGTGGCGTCCAGCTGGCAGACCATTACCAGCAGAACACCCCGATTGGTGACGGCCCGGTTCTGCTGCCGGACAACCACTATCTGTCCACTCAGAGCAAACTGTCCAAAGATCCGAACGAGAAACGCGACCACATGGTGCTGCTGGAATTTGTTACCGCTGCTGGCATCACTCTGGGTATGGATGAGCTGTACAAACTCGAGCACCACCACCACCACCACTGA | mTurquoise2 with an N-terminal 13 residue SpyTag and C-terminal hexahistidine tag was cloned into pBbA2k |
| pARH365 | pBbA2k::_ST_SYFP2-6xHis | ATGGGCGAGTTGGCTCACATTGTTATGGTTGACGCATATAAGCCGACAAAGGGAAGTGTTTCTAAAGGCGAAGAACTGTTCACGGGCGTAGTGCCAATCCTGGTGGAGCTGGATGGTGACGTGAATGGTCACAAATTCTCCGTTTCTGGTGAAGGTGAGGGTGACGCGACTTACGGCAAACTGACCCTGAAACTGATCTGCACTACCGGTAAACTGCCGGTGCCGTGGCCGACCCTGGTGACCACCCTGGGCTATGGCGTTCAGTGCTTCGCGCGTTACCCTGATtATATGAAGCAGCATGATTTCTTCAAAAGCGCGATGCCGGAAGGCTACGTGCAGGAACGTACCATCTTCTTTAAAGATGATGGCAATTACAAAACCCGTGCTGAGGTAAAATTCGAAGGTGATACCCTGGTGAACCGCATCGAACTGAAAGGCATCGATTTCAAAGAGGATGGTAACATCCTGGGTCATAAACTGGAATATAACTACAACTCCCATAACGTATATATTACGGCGGATAAACAGAAGAACGGTATTAAAGCTAACTTCAAAATCCGCCACAACATCGAGGACGGTGGTGTACAGCTGGCCGATCACTATCAACAGAATACCCCAATCGGTGACGGCCCGGTTCTGCTGCCAGATAACCACTACCTGAGCTACCAGTCCAAACTGTCTAAAGATCCAAATGAAAAACGCGACaATATGGTTCTGCTGGAGTTCGTAACCGCGGCTGGTATCACCCTGGGTATGGACGAACTGTATAAGCTCGAGCACCACCACCACCACCACTGA | mTurquoise2 from pARH364 was replaced with an *E. coli* codon-optimized version of SYFP2 (Integrated DNA Technologies, Coralville USA;^10^) using Gibson assembly. |
| pARH389 | pBbA2k::SpyTag-mTurquoise2-tev-4xC-6xH (“pProbe”) | ATGGGCGAGTTGGCTCACATTGTTATGGTTGACGCATATAAGCCGACAAAGGGAAGTGTTAGCAAAGGCGAAGAACTGTTCACCGGTGTAGTGCCGATCCTGGTTGAGCTGGATGGCGACGTGAATGGCCACAAATTCAGCGTGTCCGGTGAAGGCGAAGGTGATGCCACGTATGGTAAGCTGACCCTGAAATTTATCTGCACCACTGGTAAGCTGCCGGTTCCGTGGCCGACGCTGGTTACGACCCTGTCCTGGGGCGTGCAGTGTTTCGCCCGCTATCCGGACCACATGAAACAGCATGACTTCTTCAAAAGCGCAATGCCGGAGGGTTACGTGCAGGAACGTACCATCTTTTTCAAGGACGACGGTAACTACAAGACTCGTGCAGAAGTGAAATTTGAAGGCGATACCCTGGTTAACCGTATTGAACTGAAAGGTATCGATTTCAAAGAAGACGGCAACATCCTGGGCCACAAACTGGAATACAATTATTTTAGCGATAACGTATACATCACCGCAGACAAACAGAAAAACGGCATTAAAGCAAACTTCAAAATCCGTCACAACATTGAAGACGGTGGCGTCCAGCTGGCAGACCATTACCAGCAGAACACCCCGATTGGTGACGGCCCGGTTCTGCTGCCGGACAACCACTATCTGTCCACTCAGAGCAAACTGTCCAAAGATCCGAACGAGAAACGCGACCACATGGTGCTGCTGGAATTTGTTACCGCTGCTGGCATCACTCTGGGTATGGATGAGCTGTACAAAGGTGGAGATTACGATATCCCAACGACCGAAAACCTTTACTTCCAGGGTTCAGGCTGTTGTCCAGGCTGCTGCGGAGGTTCACTCGAGCACCACCACCACCACCACTGA | Inverse PCR was used to introduce the canonical TEV protease cleavage site and the tetracysteine FlAsH binding site (CCPGCC), both flanked by poly-G/S linkers using pARH364 as template. |

**Supplementary References**

1 Tropea, J. E., Cherry, S. & Waugh, D. S. Expression and purification of soluble His(6)-tagged TEV protease. *Methods Mol Biol* **498**, 297-307, doi:10.1007/978-1-59745-196-3_19 (2009).

2 Pettersen, E. F. *et al.* UCSF Chimera-a visualization system for exploratory research and analysis. *J Comput Chem* **25**, 1605-1612 (2004).

3 Kleffner, R. *et al.* Foldit Standalone: a video game-derived protein structure manipulation interface using Rosetta. *Bioinformatics* **33**, 2765-2767, doi:10.1093/bioinformatics/btx283 (2017).

4 Sutter, M., Greber, B., Aussignargues, C. & Kerfeld, C. A. Assembly principles and structure of a 6.5-MDa bacterial microcompartment shell. *Science* **356**, 1293-1297, doi:10.1126/science.aan3289 (2017).

5 Lassila, J. K., Bernstein, S. L., Kinney, J. N., Axen, S. D. & Kerfeld, C. A. Assembly of robust bacterial microcompartment shells using building blocks from an organelle of unknown function. *J Mol Biol* **426**, 2217-2228, doi:10.1016/j.jmb.2014.02.025 (2014).

6 Lee, T. S. *et al.* BglBrick vectors and datasheets: A synthetic biology platform for gene expression. *J Biol Eng* **5**, 12, doi:10.1186/1754-1611-5-12 (2011).

7 Gibson, D. G. *et al.* Enzymatic assembly of DNA molecules up to several hundred kilobases. *Nat Methods* **6**, 343-345, doi:10.1038/nmeth.1318 (2009).

8 Li, L., Fierer, J. O., Rapoport, T. A. & Howarth, M. Structural analysis and optimization of the covalent association between SpyCatcher and a peptide Tag. *J Mol Biol* **426**, 309-317, doi:10.1016/j.jmb.2013.10.021 (2014).

9 Goedhart, J. *et al.* Structure-guided evolution of cyan fluorescent proteins towards a quantum yield of 93%. *Nat Commun* **3**, 751, doi:10.1038/ncomms1738 (2012).

10 Kremers, G. J., Goedhart, J., van Munster, E. B. & Gadella, T. W., Jr. Cyan and yellow super fluorescent proteins with improved brightness, protein folding, and FRET Forster radius. *Biochemistry* **45**, 6570-6580, doi:10.1021/bi0516273 (2006).
